# Post-mastectomy pain syndrome in low- and middle-income countries: A systematic review

**DOI:** 10.4102/phcfm.v18i1.5350

**Published:** 2026-10-12

**Authors:** Oluwayemisi E. Ekor, Mustapha Amoadu, Sean Chetty

**Affiliations:** 1 Department of Anaesthesiology and Critical Care, Faculty of Medicine and Health Sciences, Stellenbosch University, Cape Town, South Africa; 2 Biomedical and Clinical Research Centre, College of Health and Allied Sciences, University of Cape Coast, Cape Coast, Ghana

**Keywords:** post mastectomy pain syndrome, post breast surgery pain syndrome, breast cancer, systematic review, low- and middle-income countries

## Abstract

**Background:**

Breast cancer, one of the most prevalent cancers worldwide, poses a significant health burden, particularly in low- and middle-income countries (LMICs). Despite advances in treatment, breast cancer survivors in LMICs face unique challenges, including post-mastectomy pain syndrome (PMPS).

**Aim:**

This systematic review synthesises evidence on PMPS prevalence, characteristics, and management in LMICs.

**Method:**

We conducted a systematic review in accordance with Preferred Reporting Items for Systematic Reviews and Meta-Analyses (PRISMA) 2020. We searched PubMed, CENTRAL, Embase, Web of Science, Global Index Medicus, and supplementary grey-literature sources for studies of PMPS conducted in LMICs. Eligible studies were screened in duplicate, data were extracted using a piloted form, and methodological quality was assessed using design-specific Joanna Briggs Institute critical checklists and the Mixed Methods Appraisal Tool for mixed-methods studies. Owing to substantial heterogeneity in study design, findings were synthesised narratively.

**Results:**

Findings revealed significant variability in its prevalence. Pain was mostly mild, located in the axilla, chest wall, and ipsilateral arm, neuropathic in nature, and exacerbated by physical exertion. Key risk factors include younger age, obesity, extensive surgical procedures, and adjuvant therapies. Treatment strategies encompass simple analgesics, nerve blocks, sympathetic denervation, and advanced techniques, highlighting a multifaceted approach to managing PMPS effectively.

**Conclusion:**

Post-mastectomy pain syndrome remains a complex challenge, requiring ongoing research to refine diagnostics, understand underlying mechanisms, and develop personalised treatments to improve patient outcomes.

**Contribution:**

This review synthesises evidence on the prevalence, characteristics, risk factors, and treatment options for PMPS, providing knowledge to guide evidence-based clinical interventions and improve patient outcomes.

## Introduction

Breast cancer is one of the most prevalent cancers worldwide, accounting for approximately 12% of all newly diagnosed cancer cases in 2020.^1^ The burden of breast cancer is particularly high in low- and middle-income countries (LMICs), where it remains a leading cause of morbidity and mortality among women. In Africa alone, Global Cancer Incidence, Mortality and Prevalence (GLOBOCAN) estimated 168 690 new cases and 74 072 deaths from breast cancer in 2020, underscoring the significant health challenge it poses.^1^ For example, in Ghana, around 2900 cases of breast cancer are reported annually, with a high mortality rate – approximately one in eight patients succumbs to the disease.^2^

Advances in breast cancer treatment, including surgery, chemotherapy and radiation, have greatly improved survival rates.^3^ For example, in high-income countries, Advances in treatment have resulted in 91% of patients surviving 5 years and 84% surviving 10 years post-diagnosis.^4^ However, these outcomes are not mirrored in LMICs, where late-stage diagnoses, limited treatment options and inadequate healthcare infrastructure continue to negatively impact survival rates.^5^ Surgical interventions such as mastectomy, breast-conserving surgery and axillary lymph node dissection are common treatments, but they can leave patients with long-term physical consequences, including chronic postoperative pain conditions like post-mastectomy pain syndrome (PMPS).^6^

Post-mastectomy pain syndrome is a chronic neuropathic pain condition that can persist for more than 3 months after surgery, significantly affecting the quality of life of breast cancer survivors.^7^ It is typically characterised by burning, stabbing or throbbing sensations in the chest, axilla or upper arm.^8^ The exact cause of PMPS is not fully understood, but factors such as nerve damage during surgery, preoperative pain and radiotherapy have been identified as contributors.^9^ The syndrome can severely limit survivors’ mobility and interfere with daily activities, adding to the emotional and physical burden of recovery.^10^

Managing PMPS is complex, with no standardised treatment protocol in place.^11^ Various pharmacological and non-pharmacological strategies, such as opioids, nerve blocks and physical therapy, have been proposed, but outcomes remain inconsistent,^11^ particularly in LMICs, where access to pain management services is limited.^12^ In high-income countries, where the syndrome has been more extensively studied, more comprehensive pain management systems are available.^9^ However, in resource-limited settings, the lack of infrastructure and trained personnel contributes to inadequate pain relief for PMPS sufferers.

Despite the growing body of research on breast cancer treatment in developing economies, evidence synthesis on PMPS remains underexplored. Existing reviews^13,14,15^ have focused on studies from high-income settings, leaving a critical gap in our understanding of PMPS in LMICs. This lack of research limits the ability of healthcare providers to effectively manage the condition in these areas using context-specific evidence, thereby impacting the long-term well-being of breast cancer survivors. Hence, this systematic review focuses on the prevalence and impact of PMPS in LMICs, as well as the characteristics of PMPS and effective treatment options available. The review seeks to inform strategies for more effective pain management for breast cancer survivors in LMICs. By understanding the prevalence of PMPS and its impact on quality of life in resource-constrained environments, healthcare providers and policymakers will be better equipped to improve postoperative care. This systematic review will not only shed light on the scope of PMPS but also propose critical insights for addressing the unmet needs of breast cancer survivors in LMICs, contributing to more comprehensive survivorship care.

## Methods

This review was conducted as a systematic review with structured narrative synthesis.^16^ Although studies of different designs were eligible, the included evidence was synthesised narratively because of substantial heterogeneity in study designs, PMPS definitions, pain measures and follow-up intervals. The recommendations included were identification of research questions, search strategy, study selection, data extraction, critical appraisal, data synthesis, results interpretation, reporting and implications and recommendations. Furthermore, the reporting of this systematic review followed the Preferred Reporting Items for Systematic Reviews and Meta-Analysis (PRISMA) guidelines.^17^ Registration of the protocol for this review was carried out in the Open Science framework with digital object identifier https://doi.org/10.17605/OSF.IO/D57SQ.

### Research questions

The following research questions guided this review:

- *What is the prevalence of PMPS in LMIC?*
- *What is the pattern of pain experienced by patients with PMPS?*
- *What are the risk factors for PMPS?*
- *What are the effective treatment options for PMPS?*

### Search strategy

A comprehensive search was conducted in the following bibliographic databases: PubMed, Cochrane Central Register of Controlled Trials (CENTRAL), Embase, Web of Science and Global Index Medicus. Supplementary searching included Google Scholar, ProQuest and manual screening of reference lists.

An additional search was conducted in Dimensions Ai, Google Scholar, Google, ProQuest and Institutional Repositories such as the World Health Organization (WHO) library. All searches were conducted using a combination of Medical Subject Headings (MeSH) terms, controlled vocabulary and keywords and supported by Boolean operators (“AND” “OR”). An initial search was conducted in PubMed (National Center for Biotechnology Information [NCBI]) and then modified for searches in other databases. The last search was done on 12 August 2024. A chartered librarian was consulted through search procedures. The search strategy conducted in PubMed is presented in Table 1.

**TABLE 1:** Search strategy for the prevalence, patterns, associated risk factors and effective treatment options.

Search to identify key words	Search strategy
#1: Search to identify post-mastectomy pain syndrome	“Post mastectomy pain syndrome” [MeSH Term] OR “post breast surgery pain” OR “post radical mastectomy pain” OR “post extended radical mastectomy pain” OR “post modified radical mastectomy pain” OR “post segmental mastectomy pain” OR “post simple mastectomy pain” OR “post subcutaneous mastectomy pain” OR “post prophylactic mastectomy pain” OR “chronic post-mastectomy pain” OR “persistent post-mastectomy pain” OR “post-surgical breast pain” OR “breast surgery-related pain” OR “surgical trauma-induced breast pain” OR “post-operative breast pain” OR “mastectomy-related pain” OR “post-surgical chest pain.”
#2: Search to identify prevalence of PMPS	“Prevalence” [MeSH Term] OR “rate” OR “percentage” OR “frequency” OR “occurrence” OR “incidence” OR “proportion” OR “epidemiology” OR “distribution” OR “commonness” OR “prevalence rate”
#3: Search to identify pattern of pain of PMPS	“Pattern” [MeSH Term] OR “severety” OR “intensity” OR “duration” OR “chronicity” OR “course” OR “nature” OR “characteristics” OR “manifestation” OR “features” OR “expression” OR “phenomenon” OR “temporal profile” OR “symptomatology” OR “clinical presentation” OR “time course” OR “longitudinal profile.”
#4: Search to identify risk factors for PMPS	“Risk factors” [MeSH Terms] OR “causes” OR “determinants” or “correlates” OR “contributors” OR “influences” OR “precipitants” OR “triggers” OR “etiologic factors” OR “associated factors” OR “contributors” OR “origins” OR “roots” OR “sources” OR “inducers” OR “antecedents.”
#5: Search for effective treatment options for PMPS	“Effective treatment” [MeSH Term] OR “successful interventions” OR “proven therapies” OR “successful management” OR “efficient remedies” OR “optimal care” OR “adequate interventions” OR “therapeutic effectiveness” OR “efficacious approaches” OR “successful strategies” OR “positive outcomes in treatment” OR “successful therapeutic modalities” OR “efficient medical interventions.”
#6: Search to identify LMIC	“Low-and middle-income countries” [MeSH Term] OR “Africa” OR “sub-Sarahan” OR “Asia” OR “South America” OR “Afghanistan” OR “Albania” OR “Algeria” OR “Angola” OR “Antigua and Barbuda” OR “Argentina” OR “Armenia” OR “Azerbaijan” OR “Bangladesh” OR “Belarus” OR “Belize” OR “Benin” OR “Bhutan” OR “Bolivia” OR “Bosnia and Herzegovina” OR “Botswana” OR “Brazil” OR “Bulgaria” OR “Burkina Faso” OR “Burundi” OR “Cabo Verde” OR “Cambodia” OR “Cameroon” OR “Central African Republic” OR “Chad” OR “China” OR “Colombia” OR “Comoros” OR “Congo” OR “Costa Rica” OR “Côte d’Ivoire” OR “Croatia” OR “Cuba” OR “Cyprus” OR “Czechia” OR “Democratic People’s Republic of Korea” OR “Democratic Republic of the Congo” OR “Djibouti” OR “Dominica” OR “Dominican Republic” OR “Ecuador” OR “Egypt” OR “El Salvador” OR “Equatorial Guinea” OR “Eritrea” OR “Eswatini” OR “Ethiopia” OR “Fiji” OR “Gabon” OR “Gambia” OR “Georgia” OR “Ghana” OR “Grenada” OR “Guatemala” OR “Guinea” OR “Guinea-Bissau” OR “Guyana” OR “Haiti” OR “Honduras” OR “Hungary” OR “India” OR “Indonesia” OR “Iran (Islamic Republic of)” OR “Iraq” OR “Jamaica” OR “Jordan” OR “Kazakhstan” OR “Kenya” OR “Kiribati” OR “Kuwait” OR “Kyrgyzstan” OR “Lao People’s Democratic Republic” OR “Latvia” OR “Lebanon” OR “Lesotho” OR “Liberia” OR “Libya” OR “Lithuania” OR “Luxembourg” OR “Madagascar” OR “Malawi” OR “Malaysia” OR “Maldives” OR “Mali” OR “Marshall Islands” OR “Mauritania” OR “Mauritius” OR “Mexico” OR “Micronesia (Federated States of)” OR “Moldova (Republic of)” OR “Mongolia” OR “Montenegro” OR “Morocco” OR “Mozambique” OR “Myanmar” OR “Namibia” OR “Nauru” OR “Nepal” OR “Nicaragua” OR “Niger” OR “Nigeria” OR “North Macedonia” OR “Oman” OR “Pakistan” OR “Palau” OR “Panama” OR “Papua New Guinea” OR “Paraguay” OR “Peru” OR “Philippines” OR “Poland” OR “Qatar” OR “Republic of Korea” OR “Republic of Moldova” OR “Romania” OR “Russian Federation” OR “Rwanda” OR “Saint Kitts and Nevis” OR “Saint Lucia” OR “Saint Vincent and the Grenadines” OR “Samoa” OR “Sao Tome and Principe” OR “Saudi Arabia” OR “Senegal” OR “Serbia” OR “Seychelles” OR “Sierra Leone” OR “Singapore” OR “Slovakia” OR “Slovenia” OR “Solomon Islands” OR “Somalia” OR “South Africa” OR “South Sudan” OR “Sri Lanka” OR “Sudan” OR “Suriname” OR “Syrian Arab Republic” OR “Tajikistan” OR “Thailand” OR “Timor-Leste” OR “Togo” OR “Tonga” OR “Trinidad and Tobago” OR “Tunisia” OR “Turkey” OR “Turkmenistan” OR “Tuvalu” OR “Uganda” OR “Ukraine” OR “United Arab Emirates” OR “United Republic of Tanzania” OR “Uruguay” OR “Uzbekistan” OR “Vanuatu” OR “Venezuela (Bolivarian Republic of)” OR “Viet Nam” OR “Yemen” OR “Zambia” OR “Zimbabwe.”
Overall search strategy	#1 and #6 “not animal” #2 AND #1 AND #6 “Not Animal” #3 AND #1 AND #6 “Not Animal” #4 AND #1 AND #6 “Not animal” #5 AND #1 AND #6 “Not animal”
Filters activated	English language; no limit on date filter.

PMPS, post-mastectomy pain syndrome; LMICs, low- and middle-income countries.

### Study selection

Established eligibility criteria served as the basis for screening articles. Establishing eligibility criteria was a crucial step in the systematic review or literature synthesis process, ensuring that the selected articles contribute meaningfully to the research objectives or research questions. The eligibility criteria are presented next:

#### Inclusion criteria

- Peer-reviewed and grey literature (thesis and dissertation) that explored the pervasiveness of PMPS, the type of pain experienced, duration of pain, risk or causative factors, treatment options, the impact of PMPS and the results pertaining to interventions aimed at preventing chronic pain following breast cancer-related surgery.
- Studies published in English language.
- Studies published online with no restrictions on date.
- Qualitative, quantitative and mixed-method studies.

#### Exclusion criteria

- Review articles, abstracts from conferences, book chapters, preprints and letters to the editor.
- Animal, cell line or culture and cadaver studies.
- Articles on PMPS in high-income countries.

Titles or abstracts and full texts were screened independently by two reviewers using prespecified eligibility criteria. Disagreements were resolved through discussion and, where necessary, adjudication by a senior reviewer. A pilot calibration exercise was undertaken before formal screening. The screening for the articles was performed in three phases. In phase 1, a referencing software, Mendeley, was used to remove duplicate records. In phase 2, the titles and abstracts of records identified as meeting the eligibility criteria were screened. The screening in phase 2 led to full-text-eligible records. In phase 3, the reference list of all full-text-eligible records was reviewed to identify additional full-text-eligible records. Furthermore, M.A. and O.E.E. independently screened the full-text records based on the eligibility criteria to determine inclusion. All discrepancies, inconsistencies and disagreements were resolved through weekly discussion and with the help of a reviewer (S.C.). Checking the reference lists of eligible full-text records was carried out to identify any additional relevant studies that may have been missed initially. Two independent research groups were engaged for screening full-text records; this enhanced the reliability of the inclusion process, and any discrepancies or disagreements were resolved through regular discussions and reviewer assistance, ensuring a thorough and consensus-based selection of studies based on the eligibility criteria. This approach strengthened the comprehensiveness and accuracy of the literature review.

To ensure blinding during the screening process, identifying information such as author names, affiliations and journal names was concealed from the screeners. This anonymisation of details helps to mitigate potential biases that could arise from knowledge of study origin or authorship, allowing for an unbiased assessment based solely on the content of the titles and abstracts. The benefits of blinding minimised the influence of preconceived notions about specific authors or journals, enhanced objectivity in the screening process and maintained the integrity of the systematic review by reducing the risk of unconscious biases affecting study selection.

### Data extraction

A data extraction form was developed and piloted using three included studies. Developing and piloting a data extraction form using three included studies ensured that the form was appropriately structured and that it captured relevant information consistently. This process helped in identifying and addressing any ambiguities or challenges in data extraction; this further enhanced the efficiency and reliability of subsequent extraction from the entire dataset. Furthermore, the pilot phase allowed refinement and optimisation of the data extraction form and contributed to the overall rigour and accuracy of the study’s data synthesis process. Data extracted include authors, year of publication, country, purpose of the study, study design, population, sample size, prevalence, patterns, risk factors, treatment options and its outcomes (see Table 2 for details). Two independent researchers extracted the data from the included studies, and inconsistencies were addressed during regular meetings and with a reviewer. Having two independent researchers (O.E.E. and M.A.) extract data from included studies added rigour to the process and enhanced the reliability of the data extraction. This approach helped to ensure the accuracy and completeness of the extracted information. Inconsistencies or disagreements between the two researchers were then resolved through regular meetings and consultation with a reviewer (S.C.), fostering transparency, accountability and maintaining the integrity of the data extraction process.

**TABLE 2:** Characteristics of the included studies.

Authors (citation)	Country	Design	Sample size	Participants’ characteristics	Surgical procedure	Pain definition	Pain instrument	Follow-up durations	Risk of bias
Alkan et al.^32^	Turkey	Cross-sectional survey	614	Female patients older than: 18 years old, with a diagnosis of breast cancer whose disease were in remission, were included. The patients who had completed adjuvant chemoradiotherapy and passed a minimum 6 months of interval after operation were selected. The patients under adjuvant trastuzumab therapy were also included.	Mastectomy	It is defined as: Chronic neuropathic pain in the mastectomy scar, residual breast tissue, axilla, ipsilateral arm or chest wall after breast surgery, which lasts beyond 6 months after surgery.	-	6 months after surgery	Low risk
Siddiqui et al.^33^	Pakistan	Cohort study	120	All adult female patients with biopsy-proven carcinoma of the breast who were scheduled for elective definitive breast cancer surgery, both mastectomy and conservation	Breast cancer surgery, both mastectomy and conservation	Persistent pain after mastectomy	Assessed with the numerical rating scale	3 to 6 months after surgery	Low risk
Lanaban et al.^31^	Philippines	Cross-sectional study	45 women	Female patients aged 18 years or older who had radical mastectomy for breast cancer were eligible to participate in this study	Radical mastectomy	Post-mastectomy pain syndrome (PMPS) is the chronic neuropathic pain in the anterior aspect of the thorax, axilla and/or upper half of the arm that persists beyond 3 months after mastectomy	Numerical rating scale	3 months after surgery	Low risk
Kakati et al.^29^	India	Longitudinal	120	Literate women undergoing mastectomy	Mastectomy with or without axillary dissection		Numerical rating scale (NRS).	6 months	Low risk
Fam et al.^48^	Egypt	Randomised controlled trials (RCT)	100	Patients with ICBN post-mastectomy	-	Pain defined as refractory, regardless of aetiology, when (1) Failure of treatment goals achievement in spite of using multiple evidence-based biomedical therapies and modalities in proper and acceptable fashion (2) Psychosocial disorders that could seriously influence pain outcome measures have been assessed and appropriately addressed	Visual analog scale (VAS), the quality-of-life scale (QOLS)	6 months through regular scheduled visits after 1 week, 4 weeks and 3 months	High risk
Cortés-Samacá et al.^23^	Columbia	Cohort study	76	Patients diagnosed with breast cancer Undergoing therapeutic oncology surgery	Therapeutic cancer surgery	Pain was characterised as somatic (myofascial), neuropathic and phantom breast pain	*n* (VAS)	Follow-up done (no specific time stated)	Moderate risk
Hetta et al.^49^	Egypt	RCT	61	Patients with PMPS	Radiofrequency (RF) thoracic sympathectomy	Chronic pain in the anterior thorax, axilla and/or upper half of the arm starting after surgery and persisting for more than 3 months	VAS	Follow-up intervals of 2 weeks, 1, 2, 3 and 6 months	Low risk
Abbas and Reyad^50^	Egypt	RCT	80	Patients with PMPS	Stellate ganglion RF therapy	PMPS, described as a chronic post-surgical pain disorder of neuropathic character following breast cancer surgery	VAS	1, 4, 12 and 24 weeks following the procedure	Low risk
Aliyev and Asik^28^	Turkey	Cross-sectional study	200	Female patients who underwent breast surgery	Partial mastectomy and excisional biopsy, total mastectomy and total mastectomy with simultaneous reconstructive surgery	Chronic postoperative pain was defined as pain localised at the operation site with a VAS score ≥ 4 at 6 months postoperatively	VAS	6th postoperative month	Low risk
Fabro et al.^30^	Brazil	Cohort study	174	Women undergoing surgical treatment for breast cancer	Mastectomy, axillary lymph node dissection	Defined as chronic pain persisting beyond the normal healing time of 3 months.	Tactile stimulus test for hyperaesthesia Tinel test for neuroma Patient reports for phantom breast pain	6 months postoperatively up to 8 months final follow-up	Moderate risk
Al-Rahmawy et al.^45^	Egypt	RCT	50	ASA I and II women between 20 and 60 years who underwent elective breast surgery and developed PMPS	Radical mastectomies	Post-mastectomy neuropathic pain persisting for more than 6 months after surgery, characterised by symptoms such as allodynia, burning pain, shooting pain or hyperalgesia.	VAS	3 months	Moderate risk
Hoseinzade et al.^40^	Iran	RCT	60	Patients with a history of simple mastectomy, lumpectomy or modified radical mastectomy for breast cancer without metastasis	Mastectomy/lumpectomy	Neuropathic pain resulting from breast cancer surgery, specifically identified by the presence of sensory symptoms like allodynia, burning pain, shooting pain or hyperesthesia	NRS	3 months	Moderate risk
Mbete^19^	Kenya	Cross-sectional study	409	Female breast cancer patients who underwent mastectomy	Modified radical mastectomy Radical mastectomy	Defined as chronic pain in the anterior aspect of the thorax, axilla and/or upper half of the arm, beginning after mastectomy or quadrantectomy and persisting for more than 6 months after the surgery	NRS	6 months post-surgery via telephone interview	Low risk
Tople and Krishnendu^58^	India	Case report	One patient	A 52-year-old female who underwent a left-sided modified radical mastectomy in 2019 and completed eight cycles of chemotherapy.	Left-sided modified radical mastectomy	Complex regional pain syndrome (CRPS), defined as post-traumatic painful conditions that chiefly occur distally and exceed the expected clinical course of the original trauma in intensity and duration.	Subjective assessment of the percentage of pain relief reported by the patient	15 days and 1 month	Low risk
Ilkhomovna^35^	Uzbekistan	Quasi-experimental design	89	Women who underwent surgery for breast cancer, aged 45 to 74 years	Radical mastectomy	Considers pain as a manifestation of post-mastectomy syndrome, including clinical variants such as neuropathic types with sensitive disorders such as paresthesias and decreased pain sensitivity.	Teletermography and electroneuromyography	2–3 years	Moderate risk
Vas et al.^47^	India	Case series	20	Patients with PMPS	Mastectomy	Chronic post-surgical pain lasting over 3 months following breast surgery, characterised by symptoms such as stinging, burning, tingling, numbness and electric shocks.	NRS, Pain DETECT (PD)	1 year	High risk
Cui et al.^34^	China	Cross-sectional study	420	Female breast cancer patients who underwent mastectomy between January 2011 and December 2016	Mastectomy	Defined for this study by three specific primary criteria: (1) neuropathic character (such as numbness, needle-like or burning sensations); (2) location in the ipsilateral axilla, arm, shoulder or chest wall and (3) duration lasting more than 3 months.	Short-form of McGill Pain Questionnaire (SF-MPQ)	3 months up to a year post-surgery	Low risk
Hanna et al.^51^	Egypt	RCT	30	Female patients with PMPS	Unilateral/single mastectomy, total mastectomy, modified radical mastectomy, nipple-sparing mastectomy and skin-sparing mastectomy	Neuropathic pain post-mastectomy surgery that has lasted for 6 months at least or more.	VAS	Final (5th) treatment session Longer-term follow-up was limited due to weak patient response to follow-up phone calls.	Low risk
Hashemian et al.^36^	Iran	Case–control study	111	Women who underwent mastectomy	Mastectomy	PMPS, identified by the presence of numbness, tingling, radiant, wound or burning pain in the axilla, arms, shoulders and chest on the treated side lasting for more than 3 months.	Self-made PMPS questionnaire	3 months	Low risk
Variawa et al.^24^	South Africa	Cross-sectional survey	92	Female breast cancer patients following general anaesthesia without regional anaesthesia	Radical mastectomy, modified radical mastectomy	Defined by three criteria: (1) Timing: persisting beyond the normal healing time of 3 months; (2) Character: typical of neuropathic pain (e.g. numbness, pins and needles, burning, tingling); and (3) Location: in the axilla, arm, shoulder or chest wall on the side of surgery.	DN4 questionnaire	Time of the interview, the duration of time since surgery for the participants ranged from 3 to 96 months	Low risk
Turan et al.^25^	Turkey	Longitudinal study	240	Women who had breast surgery	Radical mastectomy (MRM), mastectomy with axillary evacuation, lumpectomy	Chronic postoperative pain was defined as having pain related to the surgical site for over 3 months following surgery, after ruling out other causes such as recurrent painful episodes	VAS	3 and 6 months	Moderate risk
Kaur et al.^37^	India	Cross-sectional survey	215	Breast cancer survivors who had undergone modified radical mastectomy (MRM) or breast conservation treatment (BCT)	Radical mastectomy	Defined as pain over the anterior chest wall, axilla or ipsilateral arm that was persistent for more than 3 months after breast cancer surgery	VAS Neuropathic Pain Symptom Inventory (NPSI) FACT-B questionnaire	3 months	Low risk
Abdelghaffar and Amer^41^	Egypt	RCT	50	Females who have under gone breast surgeries	Mastectomy	Chronic post-surgical pain following mastectomy that has persisted for more than 6 months	Numeric rating scale (NRS)	3 months	Low risk
Dayem et al.^46^	Egypt	RCT	60	Patients with post-mastectomy neuropathic pain lasting more than 6 months after modified radical mastectomy	Radical mastectomies	Post-mastectomy neuropathic pain that has persisted for more than 6 months after surgery	VAS Goniometry	3 months	Moderate risk
Ahmed et al.^59^	Egypt	Cohort study	10	Patients who have had mastectomy	Mastectomies	Defined for this study as constant chronic pain lasting longer than 3 months, confined to the axilla or chest wall and certainly beginning after surgery or radiation	McGill Pain Questionnaire–Short-Form (MPQ-SF), Numeric rating scale (NRS), VAS, Brief Pain Inventory (BPI), Pain Disability Index (PDI), Neuropathic Pain Scale (NPS), FACT GOG-NTX and the Wong-Baker Faces Pain Scale	-	Moderate risk
Reyad et al.^42^	Egypt	RCT	200	Patients with breast cancer scheduled for elective breast cancer surgery	Modified radical mastectomy or conservative breast surgery	Post-mastectomy neuropathic pain involving the anterior aspect of the chest, axilla and/or upper arm with features such as numbness, tingling, burning, shooting, stinging or stabbing and hyperesthesia. If this pain persisted continuously or intermittently for more than 3 months, it was considered PMPS.	VAS Grading System for Neuropathic Pain (GSNP)	24 weeks	Low risk
Arslan et al.^26^	Turkey	Cohort	99	Patients who had undergone a mastectomy operation for breast cancer by the same surgeons	MRM, radical mastectomy, breast-conserving surgery (BCS) with axillary dissection (AD)	Pain was defined as any pain associated with the surgical intervention that ‘started and continued after mastectomy operation’.	Pain questionnaire Leeds assessment of neuropathic symptoms and signs (LANSS) pain scale	2 months	Low risk
Mohamed et al.^43^	Egypt	RCT	90	Women aged 30 to 60 years (American Society of Anesthesiologists physical status I–III) undergoing modified radical mastectomy with axillary dissection for breast carcinoma	Radical mastectomies with axillary dissection	Chronic PMPS, specifically assessed as neuropathic pain occurring after the initial recovery period.	VAS LANSS pain scale	3 months	Low risk
Khan et al.^20^	Pakistan	Cohort study	97	Female patients operated for breast cancer	Total mastectomy Conservation surgery and axillary lymph node dissection	Defined as chronic pain in the chest, armpit, upper arm and shoulder lasting more than 3 months after surgery. For the study analysis, it specifically required pain of more than 3 months with features of neuropathy.	VAS DN4 (Douleur Neuropathique 4)	-	High risk
Beyaz et al.^39^	Turkey	Cross-sectional study	131	Patient who had breast cancer surgery	Radical mastectomy	PMPS, defined as chronic neuropathic pain located in the axilla, arm, shoulder, chest wall, scar tissue or breast that has continued for at least 3 months after breast surgery and is not caused by infection.	VAS Short-Form McGill Pain Questionnaire (SF-MPQ) Douleur Neuropathique-4 (DN-4)	3 months	Low risk
Couceiro et al.^22^	Brazil	Cross-sectional	250	Women submitted to surgical treatment for breast cancer	Radical mastectomy Quadrantectomy with axillary lymphadenectomy	PMPS, defined as pain starting after mastectomy or quadrantectomy located in the anterior surface of the chest, axilla, shoulder or the upper half of the arm and persisting for longer than 3 months after the surgical procedure	VAS and the McGill pain questionnaire	3 months	Low risk
Odynets et al.^53^	Ukraine	RCT	115	Women with post-mastectomy pain	Radical mastectomy	-	VAS McGill Pain Questionnaire (MPQ)	6 months and at 12 months	Low risk
Jayasinghe and Nandasena^21^	Sri Lanka	Cross-sectional study	360	Female breast cancer survivors who had undergone mastectomy at least 3 months prior to data collection	Mastectomy	PMPS, defined as perceived pain at least 3 months after mastectomy located over the surgical scar, anterior chest wall, ipsilateral arm, or ipsilateral shoulder, with a nature of aching, burning, shooting or throbbing	Numerical pain scale	-	Low risk
Jayasinghe and Nandasena^38^	Sri Lanka	Cross-sectional study	360	Female patients who had undergone a mastectomy	Mastectomy	PMPS was defined as ‘perceived’ pain persisting for at least 3 months after mastectomy.	Numerical pain scale	16.4 months	Low risk
Meijuan et al.^27^	China	Cross-sectional	225	Women who had undergone surgery for breast cancer between January 2010 and December 2011	Mastectomy	PMPS, defined as chronic pain in the anterior aspect of the thorax, axilla and/or upper half of the arm beginning after mastectomy or quadrantectomy and persisting either continuously or intermittently for more than 3 months after surgery.	McGill Pain Questionnaire (SF-MPQ)	1 to 3 years	Low risk
Amr et al.^44^	Egypt	RCT	150	Female patients scheduled for breast cancer surgery (ASA physical status ≤ II)	Partial or radical mastectomy with axillary dissection	Chronic pain 6 months after surgery as a consequence of treatment	VAS	6 months	Low risk
Ren et al.^54^	China	Cross-sectional	1790	Female patients with breast cancer who underwent surgery under general anaesthesia between 2017 and 2021	BCS, total mastectomy	PMPS, defined as chronic pain (dull dragging, numbness, shooting or piercing) persisting for 3 months or more post-surgery, affecting the breast, chest wall, axilla or medial arm.	NRS	6 months	Low risk
Gong et al.^52^	China	Cross-sectional study	1983	Female patients confirmed with breast cancer through pathology who underwent surgical resection between February 2012 and November 2017	Total mastectomy	Defined by two primary criteria: (1) Timing: pain duration exceeding the normal tissue healing time of 3 months after breast surgery; and (2) Location: pain located at the ipsilateral armpit, arm, shoulder or chest wall.	NRS	1 year	Low risk

Note: Please see the full reference list of the article Ekor OE, Chetty S, Amoadu M. Post-mastectomy pain syndrome in low- and middle-income countries: A systematic review. Afr J Prm Health Care Fam Med. 2026;18(1), a5350. https://doi.org/10.4102/phcfm.v18i1.5350, for more information

ICBN, intercostobrachial nerve; ASA, American Society of Anesthesiologist.

Data extractors were blinded during the extraction process. Blinding the data extractors involved concealing certain information about the studies to prevent bias during the extraction process. The data extractors were blinded by removing any information that reveals the authorship, journal name or other potential identifiers from the articles or documents being reviewed. This ensured that the extractors were unaware of the specific details that could introduce bias based on study origin or author reputation. Blinding data extractors was to minimise the risk of subjective biases that may arise because of preconceived notions about certain authors, journals or study sources. By keeping the extractors unaware of these details, the process becomes more objective, impartial and focused on the content and relevance of the data to the research question. Extracted data are in Online Appendix 1.

### Critical appraisal

Methodological quality was assessed using design-specific Joanna Briggs Institute (JBI) critical appraisal checklists (including the JBI Checklist for Randomised Controlled Trials [RCTs], cross-sectional studies, cohort studies and case–control studies, as applicable). Mixed-methods studies were appraised using the Mixed Method Appraisal Tool (MMAT).^18^ Two reviewers independently appraised each study, with disagreements resolved by consensus. For ease of presentation, studies were grouped as lower, moderate or higher risk of bias on the basis of the number and importance of unmet appraisal criteria; detailed item-level judgements are provided in Online Appendix 2.

Authors employed these tools to ensure the quality, reliability and credibility of the selected studies. By employing established appraisal methodologies, the reviewer was able to provide a comprehensive understanding of the strengths and weaknesses of the gathered evidence. It is important to note that the purpose of this appraisal was not to exclude studies but rather to offer readers the opportunity to understand the quality of the papers under review. Through this process, authors sought to transparently communicate the robustness of the existing literature, as this will enable readership to gauge the reliability and trustworthiness of the evidence synthesised for our review. Appraisal was done independently by O.E.E. and M.A. and reviewed by S.C. In this systematic review of mixed-method studies, the quality appraisal was conducted using predefined risk categories for different study designs. Cross-sectional surveys were assessed and categorised as low risk (scores 6–8), moderate risk (scores 4–5) or high risk (scores < 4). RCTs were classified as low risk (scores 10–13), moderate risk (scores 7–9) or high risk (scores 1–6). Mixed-method studies were evaluated and categorised as low risk (scores 4–5), moderate risk (score 3) or high risk (scores 1–2). Case–control studies were appraised and classified as low risk (scores 1–4), moderate risk (scores 5–7) or high risk (scores 8–10). Cohort studies were appraised as low risk (scores 8–11), moderate risk (scores 5–7) and high risk (scores 1–4). See details of appraisal results in Online Appendix 2.

### Data synthesis and reporting

In the synthesis phase, quantitative findings from both quantitative and mixed-method studies were qualitised. This allowed for a nuanced understanding of the prevalence, patterns, risk factors and treatment options for PMPS in LMICs. Converging data from both quantitative and qualitative studies will provide a comprehensive perspective, enriching the synthesis process. Simultaneously conducting thematic analysis will allow for the identification and exploration of recurrent themes across studies. This approach enhanced the interpretative depth of the synthesis by capturing the multifaceted aspects of PMPS, including its prevalence, patterns, associated risk factors and effective treatment options. Thematic analysis promotes a holistic understanding and uncovers nuances that might be overlooked in a strictly quantitative or qualitative synthesis.

Narrative reporting was employed to articulate the synthesised evidence cohesively. This narrative approach ensured that the findings were presented in a contextually rich and accessible manner, facilitating comprehension for a diverse audience, including healthcare professionals, researchers and policymakers. Combining quantitative and qualitative data enable a holistic understanding of PMPS, as quantitative studies provide numerical prevalence rates, while qualitative studies offer nuanced insights into patients’ experiences, contributing to a comprehensive narrative. Thematic analysis allows for the exploration of overarching themes, providing a deeper understanding of the contextual factors influencing PMPS. This approach facilitated a comprehensive exploration of the complexity surrounding the syndrome.

Narrative reporting offered a platform for rich interpretation and storytelling, making the findings more accessible to a broader audience. This approach enhances the translational impact of the research by presenting the synthesised evidence in a compelling and meaningful manner. Inclusion of both quantitative and qualitative evidence ensured the synthesis of diverse perspectives and methodologies. This inclusive approach acknowledges the multifaceted nature of PMPS and enhances the credibility and applicability of the review’s findings. The synthesised evidence, presented through thematic analysis and narrative reporting, will effectively inform clinical practice, policy decisions and future research directions. This approach ensures that the review’s outcomes are not only scientifically robust but also practically relevant and applicable in real-world healthcare settings.

## Review findings

Search conducted in the five main databases produced 550 records. Additional 71 records were produced from the search conducted in the additional databases. In all, 621 records were produced from the search conducted. A total of 112 records were removed using the Mendeley Software. Thereafter, 509 titles and abstracts were screened by the trained graduate students, and 475 records were removed. Thus, 34 full-text records were produced from the screening of titles and abstracts. Checking the reference list led to the discovery of additional 13 eligible records. Finally, 47 full-text records were screened against the eligibility criteria. Twelve (*n* = 12) records were excluded, and 35 records were included in this systematic review (see Figure 1 for details).

**FIGURE 1:**
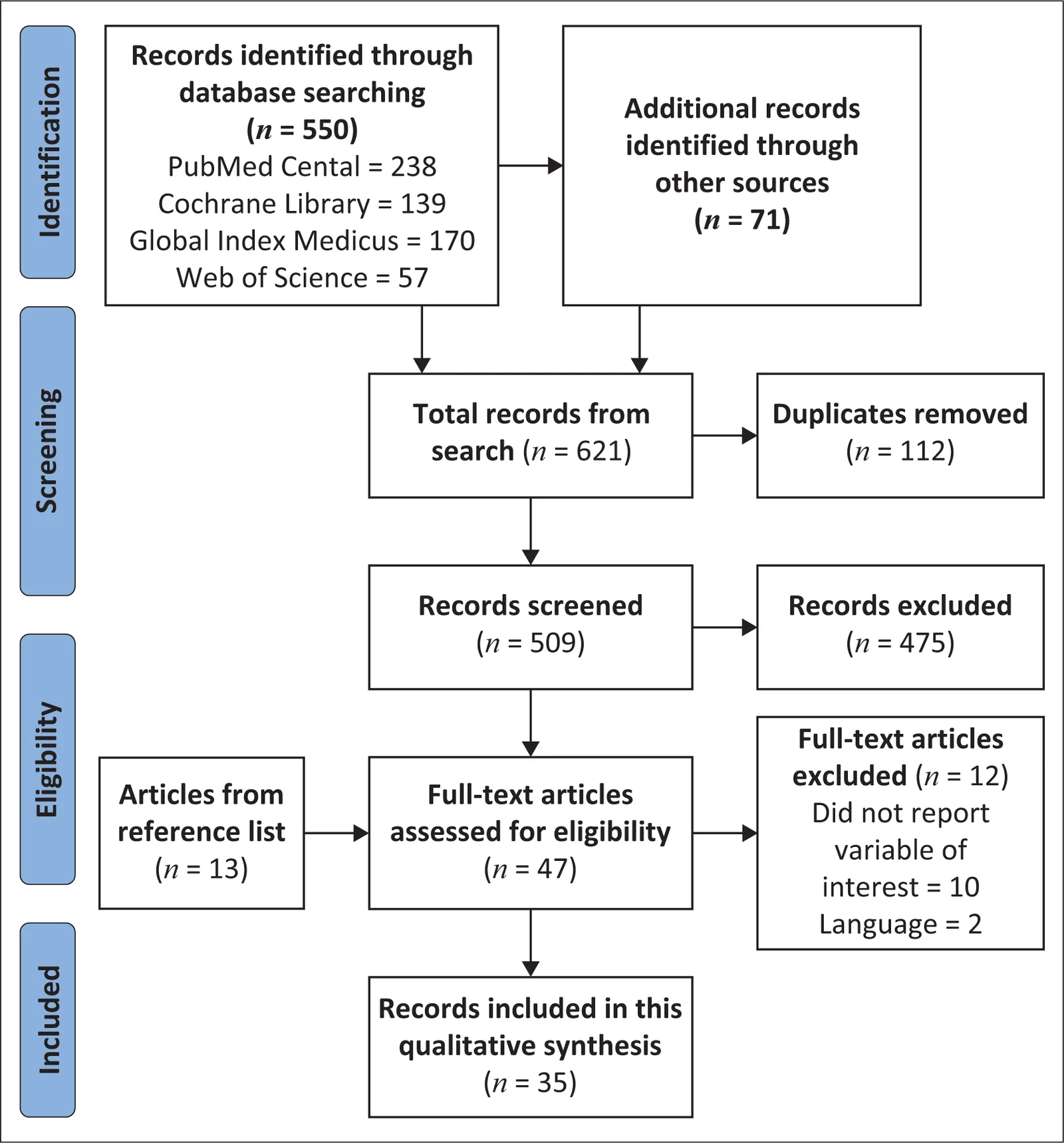
Flow chart that summarises the search results and screening process.

The included studies spanned a wide publication period from 2008 to 2024. As illustrated in Figure 2, the number of publications on PMPS varied across years, with the highest number recorded in 2018 (8 studies), followed by 2016 and 2021 (5 studies each). Figure 3 presents the distribution of study designs, showing that RCTs and cross-sectional studies were the most frequently used designs, with 13 studies each. Cohort studies were also common, accounting for 7 studies, while mixed-methods and case–control designs were less represented, with one study each. Figure 4 depicts the geographical distribution of the included studies, indicating that Egypt contributed the largest number (11 studies). Other countries, including Brazil, India, Iran and Turkey, contributed fewer studies, generally ranging from 1 to 10.

**FIGURE 2:**
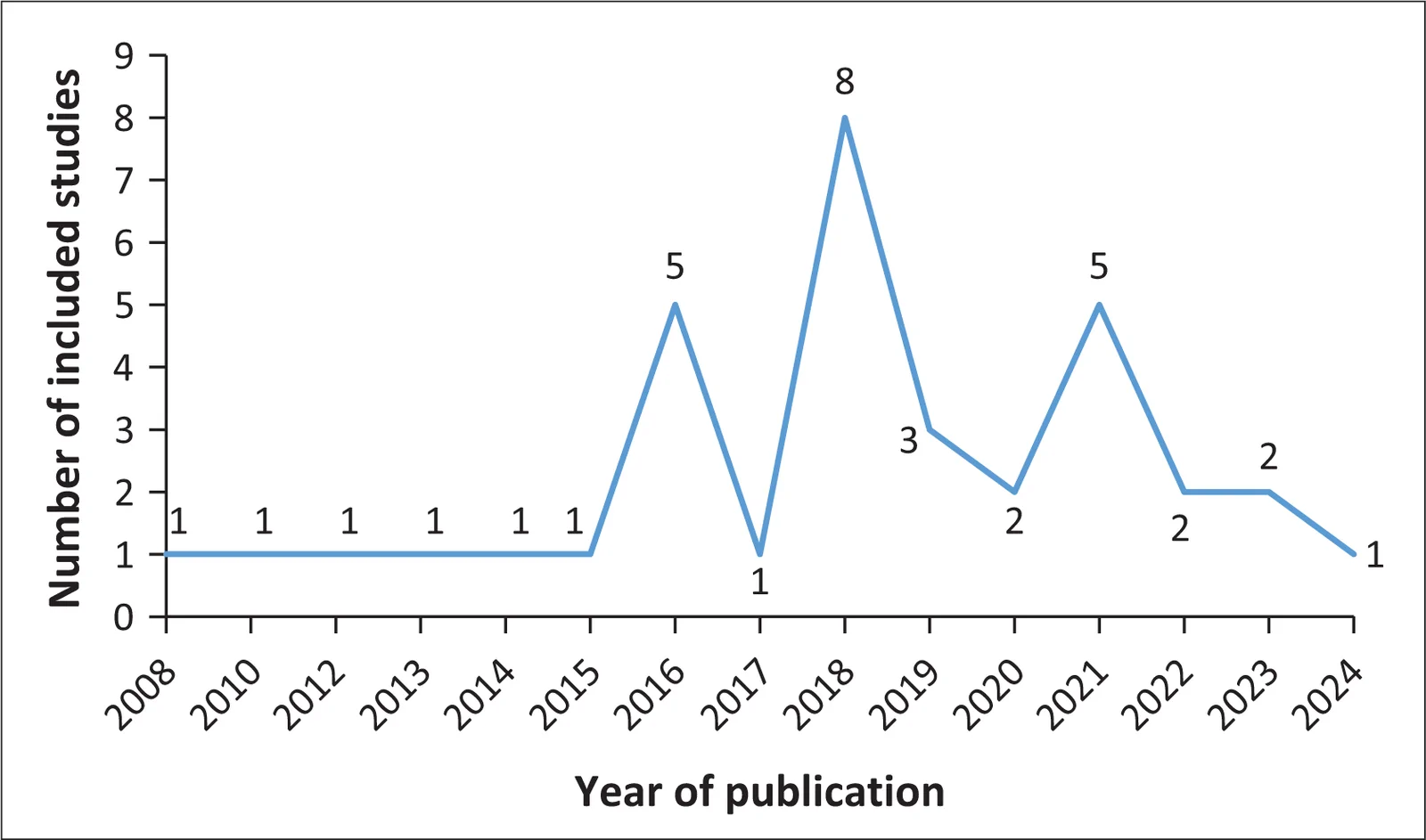
Showing the year of publication of the included studies.

**FIGURE 3:**
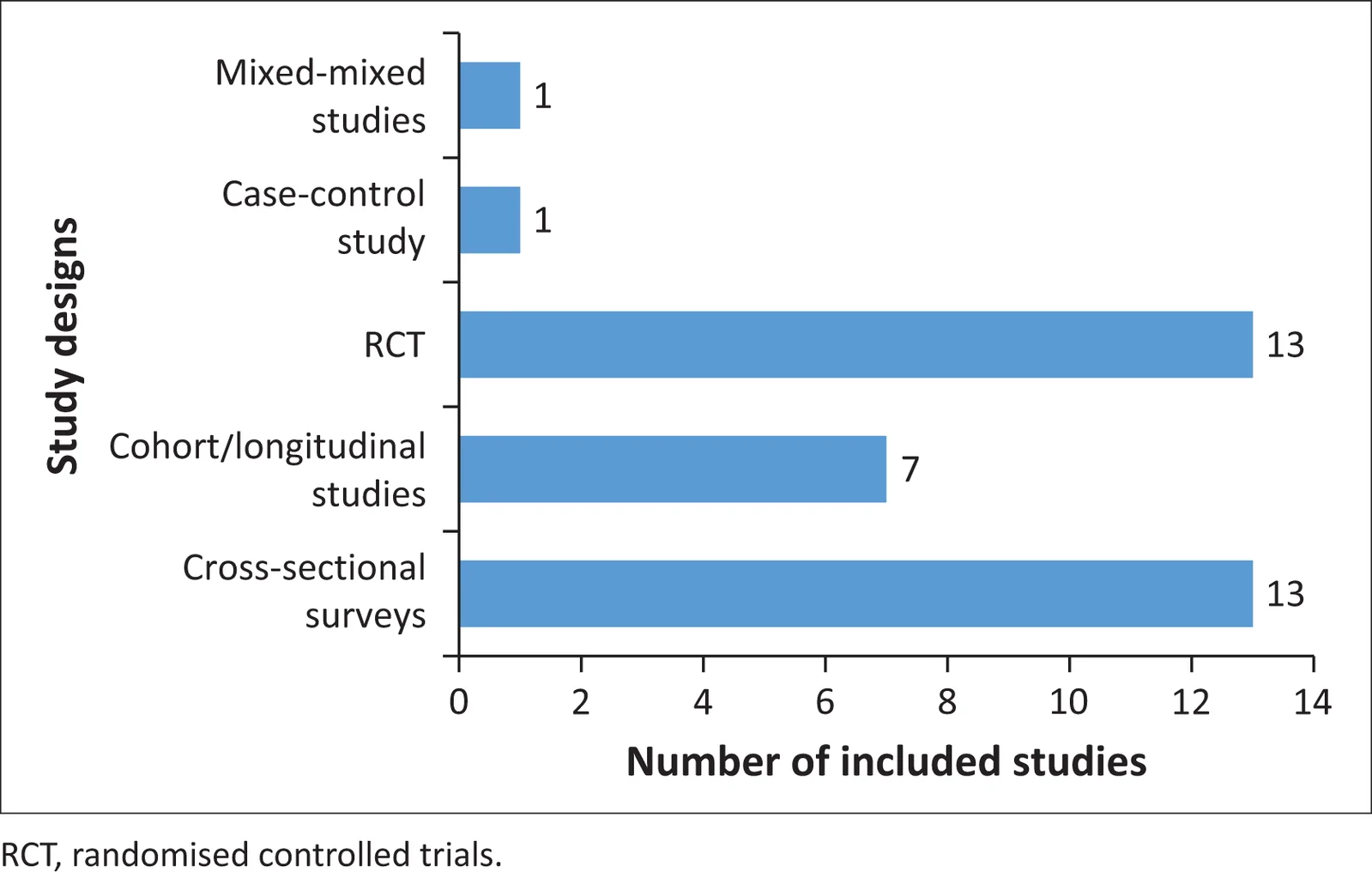
Showing the study designs of the included studies.

**FIGURE 4:**
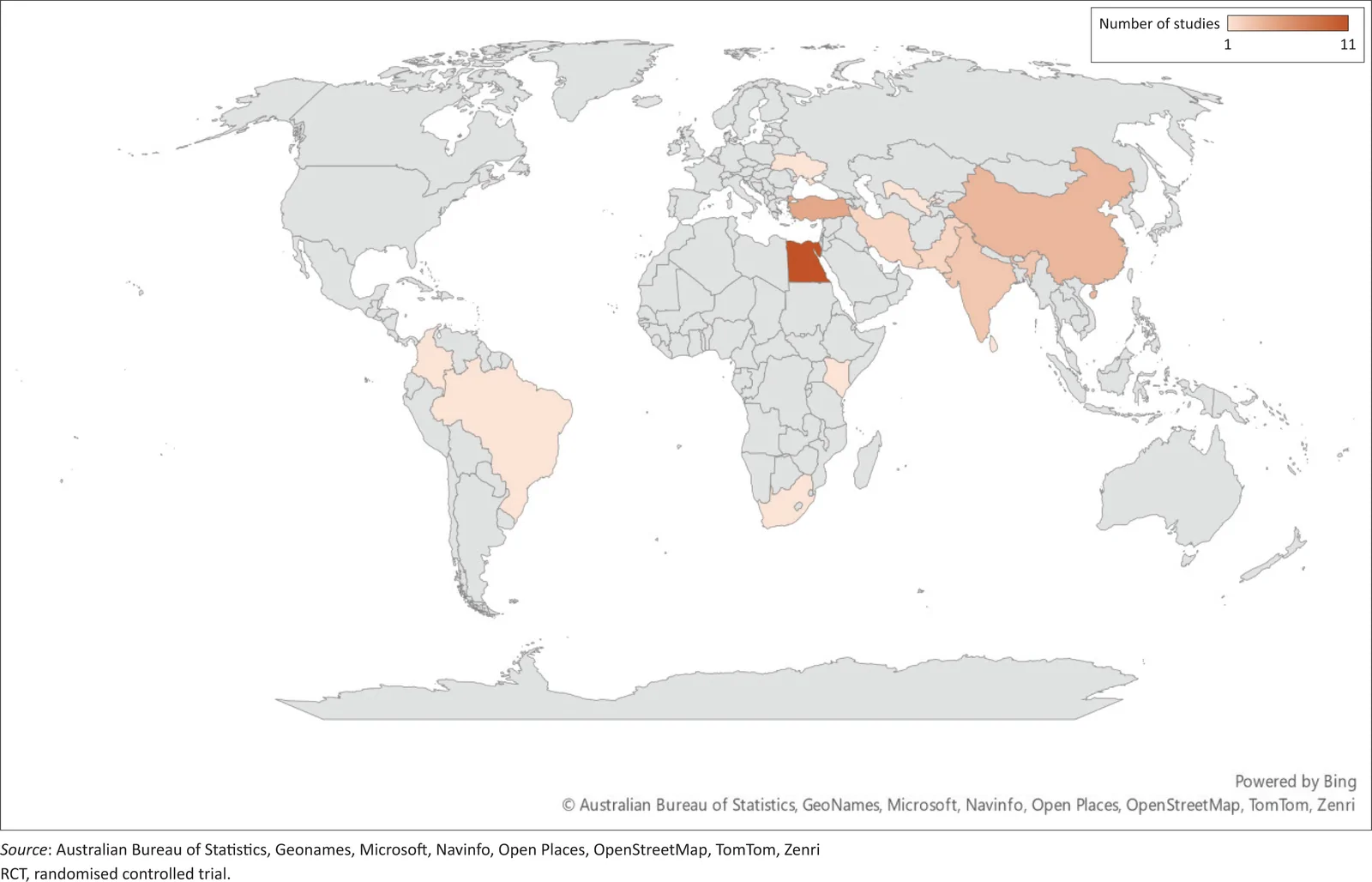
The global distribution of the included studies.

### Appraisal results

Of the included studies, cross-sectional studies generally showed fewer methodological concerns than interventional studies, whereas the RCTs varied in quality, with concerns most commonly related to allocation procedures, blinding and incomplete outcome reporting. Cohort studies showed moderate risk of bias overall, largely because of limited control for confounding and incomplete follow-up. Study-level appraisal results are presented in Online Appendix 2.

### Prevalence of post-mastectomy pain syndrome in low- and middle-income countries

The prevalence of PMPS across the included studies ranged from 14.40% to 44.40%, indicating considerable variability across settings. The lowest prevalence (14.40%) was reported in a cross-sectional study by Mbete,^19^ while the highest prevalence (44.40%) was observed in a cohort study by Couceiro et al.^22^ Other estimates were reported in predominantly cross-sectional studies, including 24.74% (Khan et al.^20^), 27.50% (Jayasinghe et al.^21^) and 42.10% (Cortés-Samacá et al.^23^), whereas Variawa et al.^24^ reported a prevalence of 38.04% using a cohort design.

### Incidence of post-mastectomy pain syndrome in low- and middle-income countries

Incidence estimates varied according to both procedure type and follow-up interval. For example, one study reported PMPS in 21.7% of patients at 3 months and 14.2% at 6 months, suggesting some decline over time, whereas another reported rates ranging from 12.5% after lumpectomy to 61.1% after lumpectomy with axillary dissection. Across studies, incidence was generally higher in patients undergoing more extensive axillary procedures and in those assessed earlier after surgery.^25,26,29,31,33^

In contrast, several studies defined PMPS based on chronic pain criteria (≥ 3 months or ≥ 6 months post-surgery) without specifying exact follow-up times. These studies reported incidence rates ranging from 24.74% to 55.3%, including 27.6% (Meijuan et al.^27^), 28.2% (Gong et al.^55^), 31.6% (Aliyev et al.^28^), 35.8% (Kakati et al.^29^), 45.1%,^32^ 52% (Fabro et al.^30^) and 55.3% (Cortés-Samacá et al.^23^). Overall, while some evidence suggests that PMPS incidence may decrease over time, comparisons across studies remain limited because of differences in follow-up duration and reporting approaches (see Table 3).

**TABLE 3:** Showing prevalence and incidence of post-mastectomy pain syndrome in low- and middle-income countries.

Themes	Rate or percentage	References
Prevalence	42.10%	^23^
	14.40%	^19^
	38.04%	^24^
	24.74%	^20^
	44.40%	^22^
	27.50%	^38^
Incidence	45.1%	^32^
	21.7% at 3 months and 14.2% at 6 months	^33^
	48.89%	^31^
	35.8%	^29^
	55.3%	^23^
	31.6%	^28^
	52%	^30^
	36.19%	^34^
	A total of 29.5% in mastectomy cases, and 64% in those having mastectomy and axillary dissection; 12.5% in patients with lumpectomy and 61.1% in those having lumpectomy and axillary dissection.	^25^
	16.2%	^26^
	27.6%	^27^
	28.2%	^52^

Note: Please see the full reference list of the article Ekor OE, Chetty S, Amoadu M. Post-mastectomy pain syndrome in low- and middle-income countries: A systematic review. Afr J Prm Health Care Fam Med. 2026;18(1), a5350. https://doi.org/10.4102/phcfm.v18i1.5350, for more information.

### Pain patterns and characteristics

Table 4 to Table 8 present data from multiple studies related to the pattern, site, character, radiation, aggravating factors, relieving factors, duration and associating factors of pain, with references to various authors. It entails the following.

**TABLE 4:** Severity of post-mastectomy pain.

Severity pattern	Description (as reported across studies)	References
Severity score	Reported pain intensity typically ranged from 1 to 10 on a numerical scale, with a median value around 3, suggesting that most patients experienced low-to-moderate pain intensity.	^32^
Predominantly mild pain	Several studies consistently reported that a majority of patients experienced mild pain (50% to 80%).	^19,21,23,26,27,29,33,39^
Moderate-to-severe pain burden	A subset of studies highlighted higher proportions of moderate or severe pain, indicating clinically significant pain levels that may require stronger management strategies.	^53,54^
Variation by activity	Pain was lower at rest but increased during movement, particularly upper limb activities such as lifting or stretching, as fewer patients reported no-to-mild pain during activity (62.9%) compared to mild pain at rest (71.2%).	^54^
VAS classification	Using VAS thresholds (0–3 = mild), most patients were classified as having mild pain, with moderate/severe pain comprising a smaller proportion.	^39^

Note: Please see the full reference list of the article Ekor OE, Chetty S, Amoadu M. Post-mastectomy pain syndrome in low- and middle-income countries: A systematic review. Afr J Prm Health Care Fam Med. 2026;18(1), a5350. https://doi.org/10.4102/phcfm.v18i1.5350, for more information.

VAS, Visual analog scale.

**TABLE 5:** Site of pain in post-mastectomy pain syndrome.

Anatomical site	Description (as reported across studies)	References
Scar, incision, mastectomy scar	Pain located on the surgical scar or mastectomy incision site; includes descriptions of pain over the residual breast area and along the surgical scar line.	^26,27,32,33,37,38^
Breast, residual breast, operated breast and nipple	Pain experienced in the breast tissue, the residual breast area or around the operated breast and nipple.	^25,27,28,32,33^
Axilla, armpit (ipsilateral)	Pain felt in the axilla/armpit on the surgical side, sometimes described as deep or radiating; often reported alongside pain in other nearby regions such as the chest wall and arm.	^8,20,26,27,28,32,33,37,39,40,52^
Chest wall (ipsilateral or anterior chest)	Pain over the anterior chest wall on the side of surgery; sometimes combined with axillary or arm pain.	^8,20,33,37,38,39,42,52^
Arm (ipsilateral, upper arm, medial upper arm)	Pain affecting the ipsilateral upper arm, medial aspect of the arm or entire arm; sometimes part of a multisite pain pattern.	^8,20,26,27,32,37,38,39,40,42,52^
Shoulder (ipsilateral)	Pain in the ipsilateral shoulder, sometimes linked with functional limitation or radiation from the chest wall or axilla.	^20,30,38,40^
Back	Pain described in the back region (less frequently reported).	^27^
Multiple or combined sites	Pain present at more than one location (e.g. axilla + breast + scar + chest wall + arm); some studies report combinations such as breast + axilla or chest wall + axilla + arm + shoulder.	^8,20,28,33,52^

Note: Please see the full reference list of the article Ekor OE, Chetty S, Amoadu M. Post-mastectomy pain syndrome in low- and middle-income countries: A systematic review. Afr J Prm Health Care Fam Med. 2026;18(1), a5350. https://doi.org/10.4102/phcfm.v18i1.5350, for more information.

**TABLE 6:** Character and type of pain.

Pain character	Description (as reported across studies)	References
Neuropathic pain	Burning, stinging, stabbing, numbness, tingling; often linked with increased sensitivity (hyperaesthesia) or pain from light touch (allodynia).	^25,28,30,31,42,47,49,50,56^
Somatic, myofascial pain	Pain from muscles or soft tissue around the surgical site.	^23^
Phantom breast pain	Pain felt in the removed breast area, even though the breast is no longer present.	^30,52^
Mixed, multiple descriptors	Many patients report more than one type of pain at the same time (e.g. burning + stabbing + numbness).	^56^
Severe functional pain	Pain scores above 6 on VAS, often with shoulder movement restriction.	^46^

Note: Please see the full reference list of the article Ekor OE, Chetty S, Amoadu M. Post-mastectomy pain syndrome in low- and middle-income countries: A systematic review. Afr J Prm Health Care Fam Med. 2026;18(1), a5350. https://doi.org/10.4102/phcfm.v18i1.5350, for more information.

VAS, Visual analog scale.

**TABLE 7:** Aggravating and relieving factors.

Factor	Description (as reported across studies)	References
Aggravating	Physical exertion, limb movement, clothing friction, tiredness, general activity	^8,32,40^
Relieving	Spontaneous regression (34%), paracetamol, NSAIDs (mild pain), opioids (severe pain), yoga, stretching	^32,33,53^

Note: Please see the full reference list of the article Ekor OE, Chetty S, Amoadu M. Post-mastectomy pain syndrome in low- and middle-income countries: A systematic review. Afr J Prm Health Care Fam Med. 2026;18(1), a5350. https://doi.org/10.4102/phcfm.v18i1.5350, for more information.

NSAID, nonsteroidal anti-inflammatory drug.

**TABLE 8:** Associated features of post-mastectomy pain.

Feature	Description (as reported across studies)	References
Neurovascular and biomechanical disorders	PMPS may be linked with underlying neurovascular involvement or functional biomechanical changes around the surgical area.	^35^
Oedema of the operated limb	Swelling of the arm, forearm or hand on the operated side is commonly observed alongside pain.	^25^
Fertility association	Women with higher fertility levels (reproductive history) were found to have a slightly lower likelihood of developing PMPS.	^36^
Radiation to the shoulder	Pain can radiate to the shoulder, potentially affecting mobility and daily activities.	^30^

Note: Please see the full reference list of the article Ekor OE, Chetty S, Amoadu M. Post-mastectomy pain syndrome in low- and middle-income countries: A systematic review. Afr J Prm Health Care Fam Med. 2026;18(1), a5350. https://doi.org/10.4102/phcfm.v18i1.5350, for more information.

PMPS, post-mastectomy pain syndrome.

#### Pain severity

Pain severity was assessed using standardised pain scales, most commonly the numerical pain rating scale (NPRS) and visual analog scale (VAS), although not all studies explicitly reported the measurement tool used. On the NPRS/VAS (0–10 scale), pain is typically categorised as mild (1–3), moderate (4–6) and severe (7–10). Across the included studies, PMPS was predominantly reported as mild in intensity. For instance, Alkan et al.,^32^ who assessed patients 6 months post-surgery, reported a median pain score of 3, corresponding to mild pain. Similarly, Siddiqui et al.^33^ (3–6 months follow-up) found that 81% of patients experienced mild pain. Kakati et al.,^29^ in a 6-month follow-up, reported that 48.8% of patients had mild pain, 32.6% moderate pain and 11.6% severe pain.

While these findings consistently indicate that PMPS is more frequently mild than moderate or severe, comparability across studies is limited because pain assessment tools and reporting approaches were not uniform, with some studies not specifying the scale used or relying on categorical classifications without clear thresholds.

#### Pain site

Pain site refers to the anatomical location where patients experience discomfort following surgery, including the surgical scar, adjacent tissues and surrounding regions such as the axilla, chest wall and ipsilateral arm. Across the included studies, the most commonly reported pain sites were the axilla, chest wall and ipsilateral arm, consistent with areas affected by surgical intervention and nerve involvement.^20,26,28,32,33,39^ For example, Alkan et al.^32^ reported that 84.1% of patients experienced pain at the scar tissue or residual breast, with additional reports of pain in the axilla and ipsilateral arm. Similarly, Siddiqui et al.^33^ identified the axilla as the most frequently reported site of pain, a finding that is consistently supported across multiple studies.^32,33,39^

#### Pain character

Neuropathic descriptors were common across studies, with burning, stabbing, throbbing, numbness, tingling, allodynia and hyperaesthesia reported most frequently. A smaller number of studies also described somatic or myofascial pain patterns and phantom breast pain.^33^ The character of pain following breast surgery varied, but neuropathic pain features were commonly reported. Siddiqui et al.^33^ described burning and throbbing sensations as prevalent among patients, indicative of neuropathic pain. Similarly, Cortés-Samacá et al.^23^ observed a combination of somatic and neuropathic pain, including symptoms such as burning and allodynia.

#### Radiation

Radiation of pain to the shoulder was observed by Fabro et al.^30^ in 27.2% of cases, indicating that post-surgical discomfort may not be confined to the breast or chest area but can extend to adjacent regions such as the shoulder.

#### Aggravating factors

Aggravating factors are activities, movements or conditions that intensify the pain experienced by patients. These could include physical exertion, posture, weather changes or stress. Physical exertion and upper limb movements were commonly cited as aggravating factors. For instance, Cui et al.^34^ found that the use of the upper limbs, particularly during tasks like lifting or stretching, significantly worsened the pain.

#### Relieving factors

Relieving factors are actions, medications or therapies that help alleviate or reduce the intensity of pain. These might include rest, medication, physical therapy or other interventions aimed at minimising discomfort. For pain relief, mild cases were generally managed with non-opioid analgesics such as paracetamol and nonsteroidal anti-inflammatory drugs (NSAIDs). More severe pain, however, required multimodal analgesic strategies, including opioids (Siddiqui et al.).^33^

#### Duration

Duration refers to the length of time patients experience pain post-surgery. It can vary from acute (lasting days to weeks) to chronic pain (persisting for months or even years). The duration of pain following breast cancer surgery varied widely among patients. Arslan et al.^26^ and Khan et al.^20^ reported that some patients continued to experience pain for several months to years.

#### Associating factors

Associated features reported across studies included oedema of the operated limb, neurovascular or biomechanical abnormalities and radiation of pain to the shoulder. One study also reported an inverse association between higher fertility history and PMPS, although the underlying basis for this finding was not explained.^25,35,36^

### Risk factors for post-mastectomy pain syndrome

Post-mastectomy pain syndrome is a persistent and often debilitating condition influenced by various factors. These factors are key contributors to its occurrence and include the following demographics, surgical procedure and its duration, administration of adjuvant therapy, along with some body symptoms.

### Demographics

Younger age, lower income and higher body mass index (BMI) were reported as factors associated with PMPS across several studies.^19,21,23,27,31,32,34,37^ However, the strength and consistency of these associations varied between studies, and most evidence came from observational designs.

### Surgical procedure and duration

More extensive surgical procedures were frequently associated with PMPS, particularly mastectomy, axillary clearance, extensive lymph node dissection, multiple drain placement, subjective lymphoedema and axillary web syndrome. Evidence for these associations was derived mainly from observational studies. Mastectomy has been identified as a key factor contributing to PMPS,^21,33^ with premastectomy conditions such as pre-existing breast pain, tumour-related pain and heightened pain sensitivity also playing a role.^31^ Axillary clearance has emerged as another significant contributor.^33,38^ Additionally, subjective lymphoedema, axillary web syndrome and total mastectomy have been observed as important contributors.^20,30^ Postoperative factors, such as the placement of two or three surgical drains, have also been associated with PMPS,^33^ as has extensive axillary lymph node dissection, particularly when more than 15 lymph nodes are removed.^30^ Furthermore, the length of time since mastectomy has been highlighted as an important risk factor.^21,31^

### Adjuvant therapy

Adjuvant therapies, particularly radiotherapy and chemotherapy, were strongly associated with the conditions under study. Alkan et al.,^32^ Siddiqui et al.,^33^ Mbete^19^ and Beyaz et al.^39^ mentioned radiotherapy, while chemotherapy was highlighted by Siddiqui et al.^33^ and Mbete.^19^

### Physical symptoms and history of psychiatric conditions

Several clinical and psychological factors were associated with an increased risk of PMPS. Lanaban et al.^31^ identified pre-existing breast pain as a significant factor, suggesting that patients who experience pain prior to surgery may be more susceptible to persistent post-surgical pain. The same study also found associations with the late-stage family life cycle although the underlying mechanism was not clearly explained. Fabro et al.^30^ reported that tissue-related factors, including the absence of tissue necrosis, were associated with PMPS, possibly reflecting differences in healing processes and nerve involvement following surgery.

A history of chronic pain was also identified as an important predictor; Cui et al.^34^ found that patients with pre-existing chronic pain conditions were more likely to develop PMPS, likely because of underlying pain sensitisation mechanisms. Similarly, Khan et al.^20^ reported that patients with larger tumours or advanced breast cancer (locally advanced breast cancer [LABC]) had a higher likelihood of experiencing PMPS, potentially because of more extensive surgical intervention and nerve damage. In addition, Beyaz et al.^39^ found that tumour localisation in the upper lateral quadrant was associated with PMPS, possibly because this region is anatomically closer to major nerves such as the intercostobrachial nerve.

Psychological factors also play a role. Alkan et al.^32^ reported that patients with post-traumatic stress disorder (PTSD) were more likely to experience PMPS, highlighting the contribution of psychological distress to pain perception and chronicity (Table 9).

**TABLE 9:** Risk factors for post-mastectomy pain syndrome.

Main theme	Sub-theme	References
Demographics	Younger age	^19,21,23,27,30,32,34,37^
	Low income	^32^
Body index	Higher BMI	^31,37^
Surgical procedure	Mastectomy	^33,38^
	Premastectomy	^31^
	Axillary clearance	^33,38^
	Two/three drain placements	^33^
	Axillary lymph node dissection (with more than 15 lymph nodes excised)	^30^
	Subjective lymphoedema	^30^
	Axillary web syndrome	^30^
	Total mastectomy	^20^
	Quadrantectomy with axillary lymphadenectomy	^22^
History of psychiatric disorders	Presence of PTSD	^32^
Adjuvant therapy	Radiotherapy	^19,32,33^
	Chemotherapy	^19,33^
Body symptoms	Breast pain	^31^
	Late-stage family life cycle	^31^
	Absence of tissue necrosis	^30^
	History of chronic pain	^34^
	Large tumour or advanced stage (LABC)	^20^
	Tumour localisation in the upper lateral quarter	^39^

Note: Please see the full reference list of the article Ekor OE, Chetty S, Amoadu M. Post-mastectomy pain syndrome in low- and middle-income countries: A systematic review. Afr J Prm Health Care Fam Med. 2026;18(1), a5350. https://doi.org/10.4102/phcfm.v18i1.5350, for more information.

BMI, body mass index; PTSD, post-traumatic stress disorder.

### Treatment options for post-mastectomy pain syndrome

Evidence for treatment was limited and came mainly from small interventional studies. Pharmacological approaches included gabapentin, pregabalin, venlafaxine, topical morphine and simple analgesics; interventional approaches included stellate ganglion block, intercostal procedures, radiofrequency-based techniques, dry needling, phototherapy and transcranial direct current stimulation (tDCS). Across these studies, outcome measures, follow-up duration and comparators varied substantially, precluding quantitative pooling. Overall, some interventions showed short-term pain reduction, but the evidence base was small, methodologically heterogeneous and insufficient to support definitive treatment recommendations.

The pharmacological management of PMPS across the included studies reflects a multimodal approach aligned with the WHO analgesic ladder, ranging from simple analgesics to centrally acting and adjuvant therapies. At the first level, non-opioid analgesics, such as paracetamol, were commonly used for mild pain. Siddiqui et al.^33^ reported that paracetamol was effective in managing mild PMPS symptoms, although its impact was limited in patients with neuropathic features. At higher levels of the analgesic ladder, adjuvant and centrally acting agents were frequently utilised. Hoseinzade et al.^40^ found that gabapentin (900 mg/day) significantly reduced pain intensity, particularly in patients with neuropathic pain. Similarly, Reyad et al.^42^ demonstrated that perioperative pregabalin (75 mg twice daily) reduced the incidence and severity of PMPS when administered from the day of surgery. Amr et al.^44^ also reported that venlafaxine 37.5 mg/day contributed to improved pain control, suggesting a role for antidepressants in modulating neuropathic pain pathways. In addition, opioid-based and localised therapies were explored. Mohamed et al.^43^ reported that topical morphine (15 mg) was associated with reduced postoperative pain, indicating its potential benefit in local pain control. Furthermore, Abdelghaffar et al.^41^ demonstrated that epidural steroid block (ESB) using a combination of bupivacaine and methylprednisolone improved pain relief and patient satisfaction, highlighting the effectiveness of interventional pharmacological approaches.

In addition to pharmacological options, nerve blocks, particularly Stellate Block (SB), have been identified as critical interventions in pain management. Al-Rahmawy et al.^45^ explored the use of magnesium, dexamethasone and lidocaine in ultrasound-guided stellate ganglion block, providing a novel approach to nerve block therapy. Furthermore, the broader application of the stellate ganglion block was studied by Dayem et al.,^46^ emphasising its role in managing pain associated with various conditions. Ultrasound-guided dry needling was reported by Vas et al.^47^ as another effective nerve block technique.

Sympathetic denervation (SD) techniques have also been explored for their efficacy in treating chronic pain conditions. Fam et al.^48^ discussed the use of pulsed radiofrequency for intercostobrachial neuralgia, highlighting its potential benefits in nerve pain management. Hetta et al.^49^ examined the effectiveness of radiofrequency thoracic sympathectomy, a procedure that targets sympathetic nerves to alleviate chronic pain. Abbas et al.^50^ compared thermal and pulsed radiofrequency of the stellate ganglion and found that thermal radiofrequency was more effective. It resulted in better pain relief, improved function and reduced need for additional pain medication over time. However, there was no difference between the two methods in terms of quality of life.

In addition, Ilkhomovna^35^ explored a combination of pharmacotherapy, manual therapy and light emitting diode (LED) phototherapy as a comprehensive approach to managing pain. More advanced interventions include tDCS, which reduces pain and increases the range of movement, as examined by Hanna et al.^51^ (Table 10).

**TABLE 10:** Effective treatment options for post-mastectomy pain syndrome.

Treatment option	Specific intervention and dosage	References
Medication	Paracetamol	^33^
	Gabapentin 900 mg per day in three divided doses	^40^
	Pharmacotherapy, manual therapy, LED phototherapy	^35^
	Transcranial direct current stimulation reduces and increases range of movement	^51^
	ESB with 30 mL (15 mL of bupivacaine 0.5%, 1 mL of 40 mg/mL of methylprednisolone, 2 mL of non-ionic contrast and 12 mL of saline 0.9%)	^41^
	The perioperative oral use of pregabalin 75 mg twice daily, starting on the morning of surgery and continued for 1 week	^42^
	Topical morphine 15 mg	^43^
	Perioperative administration of Venlafaxine 37.5 mg/day	^44^
Nerve blocks	Magnesium, dexamethasone and lidocaine in ultrasound-guided stellate ganglion block	^45^
	Ultrasound-guided dry needling	^57^
	Stellate ganglion block using (10 mL of 0.25% bupivacaine, 10 mL of 0.25% bupivacaine – 0.5 mg/kg ketamine and 10 mL of 0.25% bupivacaine – 1 mg morphine) in three groups	^46^
Other procedures	Pulsed radiofrequency for intercostobrachial neuralgia	^48^
	Radiofrequency thoracic sympathectomy	^49^
	Thermal versus super voltage pulsed	^50^
	Radiofrequency of the stellate ganglion	^50^

Note: Please see the full reference list of the article Ekor OE, Chetty S, Amoadu M. Post-mastectomy pain syndrome in low- and middle-income countries: A systematic review. Afr J Prm Health Care Fam Med. 2026;18(1), a5350. https://doi.org/10.4102/phcfm.v18i1.5350, for more information.

ESB, epidural steroid block; LED, light emitting diode.

## Discussion

### Summary of findings

Most evidence on prevalence, pain characteristics and associated factors came from cross-sectional and cohort studies, whereas treatment evidence was derived from a smaller number of RCTs and interventional studies with variable methodological quality. Accordingly, confidence in estimates of burden is moderate, but confidence in comparative treatment effectiveness remains limited. Neuropathic pain, such as burning and throbbing, is prevalent, with physical exertion exacerbating symptoms. Key risk factors include younger age, low socioeconomic status or income, higher BMI, extensive surgical procedures and adjuvant therapies such as radiotherapy and chemotherapy. Treatment strategies included non-opioid analgesics, adjuvant medications (such as gabapentin, pregabalin and venlafaxine), opioids (e.g. morphine), as well as interventional approaches including nerve blocks and SD and advanced techniques such as tDCS, highlighting a multimodal approach to managing PMPS.

### Prevalence and incidence of post-mastectomy pain syndrome in low- and middle-income countries

The wide variation in reported prevalence and incidence probably reflects differences in PMPS definitions, surgical populations, follow-up intervals and pain assessment methods across studies. Because the included studies were heterogeneous and mostly observational, direct comparison between estimates should be interpreted cautiously. For instance, Mbete^19^ reported the lowest prevalence at 14.40%, while studies by Khan et al.^20^ and Jayasinghe et al.^21^ found slightly higher rates of 24.74% and 27.50%, respectively. Conversely, Couceiro et al.^22^ documented the highest prevalence at 44.40%, with similar rates observed by Cortés-Samacá et al.^23^ at 42.10% and Variawa et al.^24^ at 38.04%. These differences can be attributed to variations in study populations, diagnostic tools and surgical techniques. For example, lower prevalence rates may stem from smaller sample sizes or different diagnostic criteria, while higher rates may involve more heterogeneous populations or greater exposure to risk factors such as more invasive surgeries or advanced stages of cancer.

Discrepancies in healthcare infrastructure, surgical practices and postoperative care across regions may also contribute to the variation in prevalence. In addition, the use of different pain assessment tools and follow-up durations may further complicate comparisons, underscoring the need for standardised methodologies in future research. A consistent approach to defining and diagnosing PMPS would enable more reliable, comparable data across contexts, helping to clarify the true burden of the condition in LMICs.

When examining incidence rates, similar variability is evident. Turan et al.^25^ reported a high incidence rate of 61.1% in patients who underwent lumpectomy with axillary dissection, highlighting the influence of more invasive procedures on postoperative pain. In contrast, lower incidence rates were reported in studies such as Arslan et al.,^26^ which may be attributed to less invasive surgical procedures or shorter follow-up periods. The highest rates reported by Cortés-Samacá^23^ and others could be because of more comprehensive follow-up and the inclusion of patients with higher-risk profiles, such as those undergoing extensive lymph node dissection.

This wide range in prevalence and incidence rates across studies reflects the complexity of PMPS, which can be influenced by a host of factors, including surgical approach, patient demographics and the healthcare context. The variability highlights the importance of adopting standardised definitions, diagnostic criteria and methodologies in PMPS research to improve the consistency and comparability of findings across different studies and settings. Standardisation would not only enhance research quality but also support better clinical management and intervention strategies in LMICs, where access to specialised care is often limited.

### Pain patterns and characteristics of post-mastectomy pain syndrome in low- and middle-income countries

Several studies reported that many patients had mild pain intensity, but clinically important moderate-to-severe pain was also described in a subset of patients. Given the heterogeneity in pain scales and reporting thresholds, pain severity should be interpreted as variable rather than uniformly mild. Pain severity across studies was generally low, with most patients experiencing mild symptoms. For example, findings from Alkan et al.^32^ (median pain score of 3/10) and Siddiqui et al.^33^ (majority reporting mild pain) consistently indicate that PMPS is more often mild rather than moderate or severe. Similarly, Kakati et al.^29^ observed that nearly half of their patients reported mild pain, with a smaller proportion experiencing moderate or severe pain. This suggests that while post-mastectomy pain is a prevalent issue, the intensity is often low. The mild nature of PMPS in most cases may reflect effective early interventions or the limited impact of the nerve damage typically associated with mastectomy procedures.^33^ However, for some patients, moderate-to-severe pain still occurs, highlighting variability in patient experiences.

The location and character of PMPS also emerged as key factors across studies. Pain is frequently reported in the axilla, chest wall and ipsilateral arm, areas that are closely associated with surgical interventions such as axillary clearance and mastectomy.^32,33^ The presence of neuropathic pain, such as burning, throbbing and allodynia, was a common finding, further emphasising the complex nature of post-surgical pain. Neuropathic pain is often difficult to manage, and its prevalence suggests that nerve damage during surgery plays a significant role in PMPS.^23^ These characteristics of pain, particularly the involvement of nerve-related symptoms, suggest that PMPS is not just a simple postoperative complication but a multifaceted condition involving both physical and neurological components.

Physical activity and upper limb movement were found to exacerbate PMPS, with patients frequently reporting increased pain during daily activities.^34^ This link between movement and pain points to the involvement of neurovascular and biomechanical factors in PMPS.^35^ The persistence of pain, lasting from months to years, further underscores the chronic nature of PMPS, with some patients reporting long-term discomfort despite interventions. This chronicity aligns with findings from other studies that highlight the role of ongoing tissue and nerve disruption in the persistence of post-surgical pain.^20^ Such prolonged pain not only impacts physical functioning but also affects the quality of life of patients, complicating recovery and rehabilitation efforts.

The variability in pain intensity, location and duration across studies indicates the heterogeneous nature of PMPS. Factors such as the extent of surgery, individual pain thresholds and pre-existing conditions may all contribute to the differences in how patients experience pain Siddiqui et al.^33^ The broad range of findings in these studies points to the complexity of PMPS, where no single factor can fully account for the variations in patient outcomes. This complexity also suggests that PMPS requires ongoing investigation to better understand the underlying mechanisms that lead to persistent pain following mastectomy.

### Risk factors of post-mastectomy pain syndrome in low- and middle-income countries

The development of PMPS in LMIC is influenced by a combination of demographic factors, surgical procedures and adjuvant therapies. The association between younger age and increased risk of PMPS may be explained by both biological and psychosocial mechanisms. Younger patients tend to have a more responsive peripheral and central nervous system, which may heighten pain sensitivity following surgical nerve injury.^32^ In addition, they are more likely to receive aggressive treatments, including extensive surgery and adjuvant therapies, which may increase the risk of nerve damage.^23,32^ Psychosocial factors may also contribute, as younger individuals often report higher levels of pain-related distress and catastrophising, which are known to amplify pain perception and contribute to the persistence of chronic post-surgical pain.

Lower income was associated with PMPS in some studies. This may reflect broader inequities in access to perioperative care and pain management, but the included studies were not designed to determine causality.

Beyond access, lower income may also be associated with delayed healthcare-seeking, reduced adherence to follow-up care and limited access to rehabilitation services such as physiotherapy, all of which can contribute to the persistence of pain. Furthermore, individuals with lower socioeconomic status often experience higher levels of psychosocial stress and reduced social support, factors that have been shown to exacerbate pain perception and increase the risk of chronic pain following surgery.^32^ These mechanisms may collectively explain the observed association between low income and increased risk of PMPS. Furthermore, a higher BMI is linked to increased risk. A higher BMI may complicate surgical outcomes and wound healing, which can compound pain and hinder recovery, contributing to the persistence of PMPS.^31,37^

Surgical factors also play a critical role in PMPS development. The extent of surgical intervention, such as mastectomy and axillary clearance, has been highlighted as a significant contributor.^21,33^ More invasive procedures, such as extensive axillary lymph node dissection, are associated with higher rates of PMPS because of greater tissue trauma and nerve involvement.^20,30^ Furthermore, the placement of surgical drains has been shown to increase the likelihood of postoperative pain.^33^ Over time, the duration since surgery has been linked to the persistence of PMPS, with nerve damage and scar tissue formation contributing to prolonged pain symptoms, as seen in studies by Lanaban et al.^31^ and Jayasinghe et al.^38^ This underscores the long-term nature of PMPS and the need for ongoing monitoring and management.

Adjuvant therapies, including radiotherapy and chemotherapy, have been implicated in exacerbating PMPS symptoms because of their effects on tissues and nerves. Radiotherapy can lead to tissue inflammation and fibrosis, while chemotherapy may contribute to nerve dysfunction, both of which can aggravate chronic pain.^19,32^ In addition, tumour characteristics, such as size and location, influence the risk of PMPS. Larger tumours or those located in sensitive areas, such as the upper lateral quadrant, can increase the likelihood of nerve involvement and postoperative pain.^39^ Psychological factors, such as the presence of PTSD, further compound the pain experience, amplifying pain perception and complicating recovery.^32^ This wide range of contributing factors highlight the complexity of PMPS in LMICs. Addressing these factors requires a multifaceted approach, considering both physical and psychological aspects to improve pain outcomes and quality of life for breast cancer survivors.

### Treatment options for post-mastectomy pain syndrome in low- and middle-income countries

The treatment options for PMPS in LMICs highlight the need for a tailored approach, given the complex and multifaceted nature of the syndrome. Simple analgesics such as paracetamol and NSAIDs are commonly used as first-line treatments because of their ability to reduce inflammation and relieve mild pain. These medications act by inhibiting cyclooxygenase enzymes (COX-1 and COX-2), which play a key role in the production of prostaglandins responsible for pain and inflammation.^32^ While effective for mild cases, these drugs may not be sufficient in addressing the neuropathic component of PMPS, where nerve damage is a central feature.^32^

Gabapentin was evaluated in included studies as a treatment option for neuropathic pain features of PMPS. It modulates the release of excitatory neurotransmitters by binding to the alpha-2-delta subunit of voltage-gated calcium channels in the central nervous system.^30^ This mechanism reduces neuronal excitability and pain transmission, making it particularly beneficial for patients experiencing neuropathic symptoms such as burning or throbbing sensations.^30^ However, because treatment studies were few and heterogeneous, its effectiveness should be interpreted cautiously.

In cases where pharmacological approaches such as gabapentin, conventional analgesics or opioids such as morphine prove insufficient, more advanced interventional techniques, such as nerve blocks, have been used. Nerve blocks, particularly stellate ganglion blocks or intercostal nerve blocks, work by temporarily interrupting the transmission of pain signals from the affected nerves to the brain.^45^ This interruption reduces the sensation of pain at the source and can provide both immediate and sustained relief for patients with persistent PMPS.^20^ The effectiveness of nerve blocks stems from their ability to target specific nerve pathways that are responsible for the chronic pain experienced in PMPS, especially in cases where surgery has damaged or sensitised these nerves.

Emerging treatments such as LED phototherapy and tDCS introduce new mechanisms for addressing PMPS, particularly for patients who have not responded to conventional treatments. LED phototherapy works by using light at specific wavelengths to promote cellular regeneration and reduce inflammation, which can be particularly helpful for managing pain associated with tissue damage and scarring following surgery.^22^ The anti-inflammatory properties of phototherapy help reduce the pain by promoting healing at the cellular level, which is crucial in cases where prolonged inflammation contributes to chronic pain.^35^

Transcranial direct current stimulation, on the other hand, offers a non-invasive neuromodulatory technique by applying a low-level electrical current to the scalp, which alters neuronal activity in the brain’s pain-processing regions.^37^ This modulation helps reduce pain perception, providing relief for patients suffering from chronic pain that persists despite pharmacological treatment.^37^ The mechanism behind tDCS is its ability to alter cortical excitability, potentially ‘resetting’ the brain’s perception of pain. This makes it particularly effective for cases where the central nervous system has become hyper-sensitised, contributing to persistent PMPS. These innovative treatments underscore the importance of incorporating both pharmacological and non-pharmacological approaches to address the complex pain mechanisms in PMPS.

### Recommendation for policy, practice and education

Given the heterogeneity of definitions and follow-up methods identified in this review, the most immediate priority for policy and practice is the adoption of standardised definitions, routine postoperative pain assessment and prospective follow-up protocols for breast surgery patients in LMIC settings.

In practice, healthcare providers are encouraged to adopt a multimodal approach to PMPS management, incorporating pharmacological treatments, nerve blocks and advanced techniques such as SD. Routine screening and early intervention for PMPS should be part of postoperative care protocols, particularly in the initial 6 months. Treatment protocols should be adapted to cultural and contextual realities, especially in LMICs, focusing on basic pain relief and cost-effective interventions. Clinicians should utilise holistic assessments that consider physical, psychological and social factors, prioritise patient-centred care and implement comprehensive post-surgical rehabilitation programmes. Embracing innovation in pain management, including new therapies and technologies, should be encouraged through clinical trials and pilot programmes.

In terms of clinical education, medical and nursing curricula should include comprehensive training on PMPS, covering its pathophysiology, risk factors and treatment strategies. Continuous education and training for healthcare professionals should focus on the latest advancements in PMPS management, including the use of standardised assessment tools. Public awareness campaigns should inform breast cancer survivors, their families and caregivers about PMPS symptoms, treatment options and the importance of early medical intervention. Support for patient advocacy groups is vital for patient education and advocacy. Regular workshops and seminars for healthcare providers should address practical aspects of PMPS management, and online knowledge exchange platforms can facilitate the dissemination of research findings and best practices, particularly in resource-limited settings.

### Limitations in this review

This review has several limitations. Firstly, substantial heterogeneity in PMPS definitions, surgical procedures, pain instruments and follow-up intervals precluded meta-analysis and limited direct comparability between studies. Secondly, most included studies were observational, which limits causal inference for associated factors. Thirdly, treatment studies were relatively few and generally small, which restricts confidence in treatment-effect estimates. Fourthly, because a meta-analysis was not undertaken, formal assessment of publication bias or small-study effects was not appropriate. Fifthly, the geographic concentration of studies in a limited number of LMIC settings reduces generalisability.

### Recommendations for future studies

Future research on PMPS should prioritise the standardisation of definitions and methodologies to enable consistent comparisons across studies, particularly in LMICs where data are sparse. In addition, longitudinal studies with extended follow-up periods are essential to capture the long-term progression of PMPS, while diverse study designs, including qualitative and cohort studies, can provide deeper insights into patient experiences and the multifaceted nature of the condition. There is also a need for more rigorous trials on interventional and pharmacological treatments, especially newer modalities such as nerve blocks and transcranial stimulation. As such, research should investigate the impact of adjuvant therapies, surgical techniques and genetic predispositions on PMPS development. Lastly, collaborative global efforts are needed to develop evidence-based clinical guidelines, and advanced imaging techniques should be utilised to better understand the anatomical and physiological aspects of PMPS.

## Conclusion

This review shows that PMPS is an important but heterogeneously reported problem in LMICs. Reported burden, pain characteristics and associated factors varied widely across studies, largely because of differences in case definitions, follow-up and study designs. Younger age, higher BMI, more extensive surgery and adjuvant therapy were recurrently associated with PMPS, but most evidence came from observational studies. Evidence for treatment effectiveness remains limited and methodologically heterogeneous. Standardised definitions, better-designed prospective studies and more rigorous interventional research are needed to strengthen the evidence base.

## References

[1] Sung VYC, Knight JF, Johnson RM, et al. Co-dependency for MET and FGFR1 in basal triple-negative breast cancers. NPJ Breast Cancer. 2021;7:1–15. 10.1038/s41523-021-00238-4 PMC7997957 33772015

[2] Osei-Afriyie S, Addae AK, Oppong S, Amu H, Ampofo E, Osei E. Breast cancer awareness, risk factors and screening practices among future health professionals in Ghana: A cross-sectional study. PLoS ONE. 2021;16:1–17. 10.1371/journal.pone.0253373 PMC8224936 34166407

[3] Cathcart-Rake EJ, Ruddy KJ. Evidence-based guidance for breast cancer survivorship. Hematol Oncol Clin North Am. 2023;37:225–243. 10.1016/J.HOC.2022.08.019 36435612

[4] Elkin EB, Hudis CA. Parsing progress in breast cancer. J Clin Oncol. 2015;33:2837–2838. 10.1200/JCO.2015.62.4890 26261261

[5] Malik M, Idrees RB, Anwar S, Kousar F, Sikandar S, Chaudhary MH. Assessing the factors leading to missed breast cancer diagnoses in mammography among Pakistani women: A prospective cross-sectional study. Cureus. 2024;16:e71436. 10.7759/cureus.71436 PMC11560408 39544607

[6] Lovelace DL, McDaniel LR, Golden D. Long-term effects of breast cancer surgery, treatment, and survivor care. J Midwifery Womens Health. 2019;64:713–724. 10.1111/jmwh.13012 31322834

[7] Vilholm OJ, Cold S, Rasmussen L, Sindrup SH. The postmastectomy pain syndrome: An epidemiological study on the prevalence of chronic pain after surgery for breast cancer. Br J Cancer. 2008;99:604–610. 10.1038/sj.bjc.6604534 PMC2527825 18682712

[8] Yi C, Li B, Pollom EL, Horst KC, Li R. Integrating radiosensitivity and immune gene signatures for predicting benefit of radiotherapy in breast cancer. Clin Cancer Res. 2018;24:4754–4762. 10.1158/1078-0432.CCR-18-0825 PMC6168425 29921729

[9] Capuco A, Urits I, Orhurhu V, et al. A comprehensive review of the diagnosis, treatment, and management of postmastectomy pain syndrome. Curr Pain Headache Rep. 2020;24(8):41. 10.1007/s11916-020-00876-6 32529416

[10] Brown MRD, Ramirez JD, Farquhar-Smith P. Pain in cancer survivors. Br J Pain. 2014;8:139–153. 10.1177/2049463714542605 PMC4616728 26516548

[11] Chappell AG, Yuksel S, Sasson DC, Wescott AB, Connor LM, Ellis MF. Post-mastectomy pain syndrome: An up-to-date review of treatment outcomes. JPRAS Open. 2021;30:97–109. 10.1016/j.jpra.2021.07.006 PMC8426165 34522756

[12] Oluwole SF, Oluwole OO. How to diagnose and treat benign and malignant diseases of the breast in low- and middle-income countries. In: Hardy MA, Hochman BR, editors. Global surgery. Cham: Springer, 2023; p. 361–380. 10.1007/978-3-031-28127-3_30

[13] Narusawa E, Sadeghi S, Tane K, Al-Khaifi M, Kikawa Y. Updates on the preventions and management of post-mastectomy pain syndrome beyond medical treatment: A comprehensive narrative review. Ann Palliat Med. 2023;13(5):1258–1264. 10.21037/apm-24-73 39168643

[14] Larsson IM, Ahm Sørensen J, Bille C. The post-mastectomy pain syndrome – A systematic review of the treatment modalities. Breast J. 2017;23:338–343. 10.1111/tbj.12739 28133848

[15] Wu RR, Katz S, Wang J, Doan L V. Prevention of post-mastectomy pain syndrome: A review of recent literature on perioperative interventions. Curr Oncol Rep. 2024;26:865–879. 10.1007/s11912-024-01553-2 38814502

[16] Stern C, Lizarondo L, Carrier J, et al. Methodological guidance for the conduct of mixed methods systematic reviews. JBI Evid Synth. 2020;18:2108–2118. 10.11124/JBISRIR-D-19-00169 32813460

[17] Page MJ, McKenzie JE, Bossuyt PM, et al. The PRISMA 2020 statement: An updated guideline for reporting systematic reviews. BMJ. 2021;372:n71. 10.1136/bmj.n71 PMC8005924 33782057

[18] Pluye P, Hong QN. Combining the power of stories and the power of numbers: Mixed methods research and mixed studies reviews. Annu Rev Public Health. 2014;35:29–45. 10.1146/ANNUREV-PUBLHEALTH-032013-182440 24188053

[19] Mbete JO. Chronic post mastectomy pain in adult female patients at Aga Khan University Hospital Nairobi (Unpublished master’s dissertation). Aga Khan University; 2019.

[20] Khan S, Nofil S, Arif A, Mir Jan M, Riaz B, Khan Sherwani NZF. Frequency and risk factors for post mastectomy pain syndrome [PMPS] in female breast cancer patients. Pak J Med Health Sci. 2021;15:2530–2533. 10.53350/pjmhs2115102530

[21] Jayasinghe V, Nandasena S. Prevalence of post mastectomy pain syndrome and its impact on quality of life of breast cancer survival in Sri Lanka. IJACM. 2018;1:11–15. 10.33545/comed.2018.v1.i3a.24

[22] Couceiro TC, Valença MM, Raposo MC, Orange FA, Amorim MM. Prevalence of post-mastectomy pain syndrome and associated risk factors: A cross-sectional cohort study. Pain Manag Nurs. 2014;15:731–737. 10.1016/j.pmn.2013.07.011 24144570

[23] Cortés-Samacá CA, Mayorga-Anaya HJ, Moreno-Ríos C, et al. Incidence of post-mastectomy pain syndrome in Colombia. Colomb J Anesthesiol. 2018;46:18–22. 10.1097/CJ9.0000000000000040

[24] Variawa ML, Scribante J, Perrie H, Chetty S. The prevalence of chronic postmastectomy pain syndrome in female breast cancer survivors. South Afr J Anaesth Analg. 2016;22:108–113. 10.1080/22201181.2016.1191214

[25] Turan M, Karaman Y, Tekgül ZT, Karaman S, Uyar M. Incidence and risk factors of chronic postoperative pain after breast surgery. Anatol J Gen Med Res. 2016;26:207–214. 10.5222/terh.2016.207

[26] Arslan E, Çermik TF, Trabulus FDC, Talu ECK, Başaran Ş. Role of 18F-FDG PET/CT in evaluating molecular subtypes and clinicopathological features of primary breast cancer. Nucl Med Commun. 2018;39:680–690. 10.1097/MNM.0000000000000856 29893750

[27] Meijuan Y, Zhiyou P, Yuwen T, Ying F, Xinzhong C. A retrospective study of postmastectomy pain syndrome: Incidence, characteristics, risk factors, and influence on quality of life. ScientificWorldJournal. 2013;2013:159732. 10.1155/2013/159732 PMC3863453 24379736

[28] Aliyev D, Asik I. Postoperative chronic pain syndrome and risk factors in patients with breast surgery. J Coll Physicians Surg Pak. 2023;33:609–615. 10.29271/jcpsp.2023.06.609 37300253

[29] Kakati B, Nair N, Chatterjee A. Post mastectomy pain syndrome at an Indian tertiary cancer centre and its impact on quality of life. Indian J Cancer. 2023;60(2):275–281. 10.4103/ijc.ijc_861_21 37530253

[30] Fabro AEN, Bergmann A, Carolina A, et al. Post-mastectomy pain syndrome: Incidence and risks. Breast. 2012;21:321–325. 10.1016/j.breast.2012.01.019 22377590

[31] Lanaban ARD, Sorrosa RJ, Concha AS. Psychosocial factors related to postmastectomy pain syndrome among women with breast cancer: Cross-sectional study. SPMC J Health Care Serv [serial online]. 2019 [cited n.d.];5(1):1. Available from: https://n2t.net/ark:/76951/jhcs5gxf88

[32] Alkan A, Guc ZG, Senler FC, Yavuzsen T. Breast cancer survivors suffer from persistent postmastectomy pain syndrome and posttraumatic stress disorder (ORTHUS study): A study of the palliative care working committee of the Turkish Oncology Group (TOG). Support Care Cancer. 2016;34(9):3747–3755. 10.1007/s00520-016-3202-6 27039206

[33] Siddiqui AS, Ahmed A, Siddiqui AS, Zeeshan S, Ahmed A, Khan S. Persistent post-surgical pain following breast cancer surgery: An observational study in a tertiary care hospital. J Pak Med Assoc. 2021;71(3):849–853. 10.47391/JPMA.946 34057934

[34] Cui L, Fan P, Qiu C, Hong Y. Single institution analysis of incidence and risk factors for post- mastectomy pain syndrome. Sci Rep. 2018;8(1):11494. 10.1038/s41598-018-29946-x PMC6068100 30065342

[35] Shikhkerimov RK, Savin AA, Welsher LZ, et al. Pathology of the Brachial Neurovascular Bundle in clinical manifestations of postmastectomy syndrome. Bull Pirogov Natl Med Surg Cent. 2011;6(4):86–90.

[36] Hashemian M, Aflatoonian M, Aflatoonian B, Esmaeil M, Akbari A. Fertility: A control factor for post mastectomy pain syndrome in breast cancer patients. Int J Cancer Manag. 2016;9(5):e5119. 10.17795/ijcp-5119

[37] Kaur N, Kumar A, Saxena AK, Gupta A, Grover RK. Postmastectomy chronic pain in breast cancer survivors: An exploratory study on prevalence, characteristics, risk factors, and impact on quality of life. Indian J Surg. 2017;80:592–598. 10.1007/s12262-017-1663-6

[38] Jayasinghe V, Nandasena S. Post mastectomy pain syndrome: A hidden facet among patients underwent mastectomy in Sri Lanka. JCCPSL. 2018;24:97. 10.4038/jccpsl.v24i2.8151

[39] Beyaz SG, Ergönenç JŞ, Ergönenç T, Sönmez ÖU, Erkorkmaz Ü, Altintoprak F. Postmastectomy pain: A cross-sectional study of prevalence, pain characteristics, and effects on quality of life. Chin Med J (Engl). 2016;129:66–71. 10.4103/0366-6999.172589 PMC4797545 26712435

[40] Hoseinzade H, Mahmoodpoor A, Agamohammadi D, Sanaie S. Comparing the effect of stellate ganglion block and gabapentin on the post mastectomy pain syndrome. Shiraz E-Med J. 2008;9:88–96.

[41] Abdelghaffar NA, Amer GF. Comparison of different volumes spread of erector spinae block in postmastectomy pain syndrome management: A prospective randomized comparative study. BMC Anesthesiol. 2023;23(1):282. 10.1186/s12871-023-02239-1 PMC10464247 37608269

[42] Reyad RM, Omran AF, Abbas DN, et al. The possible preventive role of pregabalin in postmastectomy pain syndrome: A double-blinded randomized controlled trial. J Pain Symptom Manage. 2019;57:1–9. 10.1016/j.jpainsymman.2018.10.496 30359684

[43] Mohamed SA, Abdel-Ghaffar HS, Kamal SM. Effect of topical morphine on acute and chronic postmastectomy pain. Reg Anesth Acute Pain. 2016;41:704–710. 10.1097/AAP.0000000000000496 27755490

[44] Amr YM, Yousef AAA-M. Evaluation of efficacy of the perioperative administration of venlafaxine or gabapentin on acute and chronic. Clin J Pain. 2010;26:381–385. 10.1097/AJP.0b013e3181cb406e 20473044

[45] Al-Rahmawy GF, Al-Moteleb EAABD, El-Ebeidy MG. Magnesium sulfate as an adjuvant in ultrasound guided stellate ganglion block for post mastectomy pain syndrome. Med J Cairo Univ. 2021;89:975–985. 10.21608/mjcu.2021.184526

[46] Dayem OTA, Saeid MM, Ismail OM, Badrawy AME, Ghaffar NAA. Ultrasound guided stellate ganglion block in postmastectomy pain syndrome: A comparison of ketamine versus morphine as adjuvant to bupivacaine. J Anesthesiol. 2014;2014:792569. 10.1155/2014/792569

[47] Vas J, Cintado MC, Aranda-Regules JM, Aguilar I, Ruiz FR. Effect of ear acupuncture on pregnancy - related pain in the lower back and posterior pelvic girdle: A multicenter randomized clinical trial. Acta Obstet Gynecol Scand. 2019;98(10):1307–1317. 10.1111/aogs.13635 31034580

[48] Fam BN, El-Din GG, El-Sayed, Reyad RM, Mansour I. Efficacy and safety of pulsed radiofrequency and steroid injection for intercostobrachial neuralgia in postmastectomy pain syndrome – A clinical trial. Saudi J Anaesth. 2018;12(2):227–234. 10.4103/sja.SJA_576_17 PMC5875210 29628832

[49] Hetta DF, Mohamed AA, Hetta HF, El-Barody MM, Mohammad MAF. Radiofrequency thoracic sympathectomy for sympathetically maintained chronic post-mastectomy pain, a preliminary report: 6-month results. Pain Pract. 2020;21(1):54–63. 10.1111/papr.12933 32629535

[50] Abbas DN, Reyad RM. Thermal versus super voltage pulsed radiofrequency of stellate ganglion in post- mastectomy neuropathic pain syndrome: A prospective randomised trial. Pain Physician. 2018;21(4):351–362. 10.36076/ppj.2018.4.351 30045592

[51] Hanna M, Perrot S, Varrassi G. Critical appraisal of current acute LBP management and the role of a multimodal analgesia: A narrative review. Pain Ther. 2023;12:377–398. 10.1007/s40122-023-00479-0 PMC10036717 36765012

[52] Gong Y, Tan Q, Qin Q, Wei C. Prevalence of postmastectomy pain syndrome and associated risk factors. Medicine (Baltimore). 2020;99(20):e19834. 10.1097/MD.0000000000019834 PMC7253604 32443289

[53] Odynets T, Briskin Y, Sydorko O, Tyshchenko V, Putrov S. Effectiveness of individualized physical rehabilitation programs on post-mastectomy pain in breast cancer survivors. Physiother Quart. 2018;26:1–5. 10.5114/pq.2018.78377

[54] Ren Y, Kong X, Yang Q, et al. Incidence, risk factors, prevention and treatment of postmastectomy pain syndrome in breast cancer: A multicenter study. Int J Surg. 2022;106:106937. 10.1016/J.IJSU.2022.106937 36152923

[55] Gong C, Wang J, Cheng Q, Ren W, Bai J. Mesenchymal stromal cells in adipose tissue of breast cancer patients: A cross-sectional study. Cell Mol Biol. 2005;51:1. 32415930

[56] Kaur E, Charan GS. Effectiveness of educational intervention on knowledge toward obligatory vaccination among mothers of under five children. IJHSR. 2022;12:25–29. 10.52403/ijhsr.20221204

[57] Lakshmi V, Pai R. Ultrasound – Guided dry needling as a treatment for postmastectomy pain syndrome – A case series of twenty patients. Indian J Palliat Care. 2019;25(1):93–102. 10.4103/IJPC.IJPC_24_18 PMC6388608 30820110

[58] Tople JP, Krishnendu S. Post mastectomy and radiation therapy: complex regional pain syndrome-a case report. J Pharm Res Int. 2021;33(60B):508–512. 10.9734/jpri/2021/v33i60B34647

[59] Ahmed OSM, Youssef L, Moaz I, Aboali SEM, Roshdy SHA. Evaluation and re-evaluation of post-mastectomy pain syndrome by breast cancer edge task force outcomes: clinical measures of pain after pain management protocol of physiotherapy. J Pharm Negat Results. 2022 [cited n.d.];13(Suppl 5):1243–1254. Available from: https://mail.pnrjournal.com/index.php/home/article/view/2908

